# Predicting hospitalisation for heart failure and death in patients with, or at risk of, heart failure before first hospitalisation: a retrospective model development and external validation study

**DOI:** 10.1016/S2589-7500(22)00045-0

**Published:** 2022-05-10

**Authors:** Joshua Bradley, Erik B Schelbert, Laura J Bonnett, Gavin A Lewis, Jakub Lagan, Christopher Orsborne, Pamela F Brown, Josephine H Naish, Simon G Williams, Theresa McDonagh, Matthias Schmitt, Christopher A Miller

**Affiliations:** a Division of Cardiovascular Sciences, School of Medical Sciences, Faculty of Biology, Medicine and Health, Manchester Academic Health Science Centre, University of Manchester, Manchester, UK; b Wellcome Centre for Cell-Matrix Research, Division of Cell-Matrix Biology and Regenerative Medicine, School of Biology, Faculty of Biology, Medicine and Health, Manchester Academic Health Science Centre, University of Manchester, Manchester, UK; c BHF Manchester Centre for Heart & Lung Magnetic Resonance Research, Manchester University NHS Foundation Trust, Manchester, UK; d Department of Medicine, University of Pittsburgh School of Medicine, University of Pittsburgh, Pittsburgh, PA, USA; e Clinical and Translational Science Institute, University of Pittsburgh, Pittsburgh, PA, USA; f UPMC Cardiovascular Magnetic Resonance Center, Heart and Vascular Institute, Pittsburgh, PA, USA; g Department of Health Data Science, University of Liverpool, Liverpool, UK; h School of Cardiovascular Medicine & Sciences, King's College Hospital, London, UK

## Abstract

**Background:**

Identifying people who are at risk of being admitted to hospital (hospitalised) for heart failure and death, and particularly those who have not previously been hospitalised for heart failure, is a priority. We aimed to develop and externally validate a prognostic model involving contemporary deep phenotyping that can be used to generate individual risk estimates of hospitalisation for heart failure or all-cause mortality in patients with, or at risk of, heart failure, but who have not previously been hospitalised for heart failure.

**Methods:**

Between June 1, 2016, and May 31, 2018, 3019 consecutive adult patients (aged ≥16 years) undergoing cardiac magnetic resonance (CMR) at Manchester University National Health Service Foundation Trust, Manchester, UK, were prospectively recruited into a model development cohort. Candidate predictor variables were selected according to clinical practice and literature review. Cox proportional hazards modelling was used to develop a prognostic model. The final model was validated in an external cohort of 1242 consecutive adult patients undergoing CMR at the University of Pittsburgh Medical Center Cardiovascular Magnetic Resonance Center, Pittsburgh, PA, USA, between June 1, 2010, and March 25, 2016. Exclusion criteria for both cohorts included previous hospitalisation for heart failure. Our study outcome was a composite of first hospitalisation for heart failure or all-cause mortality after CMR. Model performance was evaluated in both cohorts by discrimination (Harrell's C-index) and calibration (assessed graphically).

**Findings:**

Median follow-up durations were 1118 days (IQR 950–1324) for the development cohort and 2117 days (1685–2446) for the validation cohort. The composite outcome occurred in 225 (7·5%) of 3019 patients in the development cohort and in 219 (17·6%) of 1242 patients in the validation cohort. The final, externally validated, parsimonious, multivariable model comprised the predictors: age, diabetes, chronic obstructive pulmonary disease, N-terminal pro-B-type natriuretic peptide, and the CMR variables, global longitudinal strain, myocardial infarction, and myocardial extracellular volume. The median optimism-adjusted C-index for the externally validated model across 20 imputed model development datasets was 0·805 (95% CI 0·793–0·829) in the development cohort and 0·793 (0·766–0·820) in the external validation cohort. Model calibration was excellent across the full risk profile. A risk calculator that provides an estimated risk of hospitalisation for heart failure or all-cause mortality at 3 years after CMR for individual patients was generated.

**Interpretation:**

We developed and externally validated a risk prediction model that provides accurate, individualised estimates of the risk of hospitalisation for heart failure and all-cause mortality in patients with, or at risk of, heart failure, before first hospitalisation. It could be used to direct intensified therapy and closer follow-up to those at increased risk.

**Funding:**

The UK National Institute for Health Research, Guerbet Laboratories, and Roche Diagnostics International.

## Introduction

The high prevalence, poor prognosis, and high cost of heart failure make it a global health priority.^1^ Admission to hospital (hospitalisation) for heart failure, in particular, portends an extremely poor prognosis, with 20–30% of patients dying in the subsequent year, has a major adverse effect on quality of life, and is expensive, accounting for the majority of costs associated with heart failure.^2^

Around a third of people aged 55 years will develop heart failure.^3^ As the population ages and risk factors for heart failure become more prevalent, identifying people who are at risk of hospitalisation for heart failure and death would potentially facilitate preventive intervention.

Existing heart failure prediction models have often been developed in groups of patients who are already known to be at high risk of adverse outcomes, such as patients who have already been hospitalised for heart failure, with relatively sparse, non-contemporary phenotyping, and without external validation.^4^ Performance of such models is limited, and none have translated into routine clinical practice. Contemporary imaging biomarkers of cardiac injury and adaptation show promise for risk stratification but have not been evaluated in conjunction with other important heart failure predictors.

**Research in context**

**Evidence before this study**

We searched MEDLINE (PubMed) for studies published in English between database inception and July 5, 2021, deriving or validating multivariable risk prediction models in patients with, or at risk of, heart failure to predict adverse outcomes, including hospitalisation for heart failure and death. The search was limited to human studies. Search terms comprised: “heart failure”, “chronic heart failure”, “acute decompensated heart failure”, “heart failure with preserved ejection fraction”, “heart failure with reduced ejection fraction”, “prognostic model”, “prognostic modelling”, “prognosis”, “risk prediction”, “risk score”, “score”, “model”, “mortality”, “death”, “(re)hospitalisation”, and “(re)admission”. Heart failure is common, has a poor prognosis, and its associated health-care costs, particularly related to hospitalisation for heart failure, are high. Identifying individuals who are more likely to be hospitalised for heart failure or die before they are hospitalised is a priority. Typically, existing risk prediction models have not been subject to external validation and were developed using a high proportion of patients who have previously been hospitalised for heart failure. Such models are applicable only later in the patient pathway when patients already have a dismal prognosis, the underlying disease mechanisms might intuitively be less modifiable, and the large expenditure associated with hospitalisation for heart failure has already been incurred.

**Added value of this study**

We present an accurate, validated, and reliable prognostic model that provides individualised estimates of the risk of hospitalisation for heart failure and all-cause mortality in patients with, or at risk of, heart failure, before first hospitalisation. The model was derived from, and validated in, large cohorts of patients by use of contemporary, deep phenotyping. The derived and validated model showed excellent performance (according to discrimination and calibration metrics), and, crucially, was developed in patients before they were first hospitalised for heart failure. To our knowledge, it is the first such model.

**Implications of all the available evidence**

The final externally validated model is intuitive and includes key comorbidities that are in keeping with previous studies, circulating biomarkers, and measures of myocardial structure and function. We present a risk calculator to facilitate individual (personalised) risk stratification, which, when implemented, could allow the direction of intensified therapy and closer follow-up to those at increased risk, with the aim of preventing, or postponing, hospitalisation for heart failure and death, while redirecting unnecessary intervention away from those at low risk. This implementation could increase health-care efficiency, reduce unnecessary medicalisation, and facilitate research into preventive strategies.

We aimed to develop and externally validate a prognostic model involving contemporary deep phenotyping with cardiovascular magnetic resonance (CMR) that can be used to generate individual risk estimates of hospitalisation for heart failure and all-cause mortality in patients with, or at risk of, heart failure, but who have not previously been hospitalised for heart failure.

## Methods

### Study population

**Figure 1:**
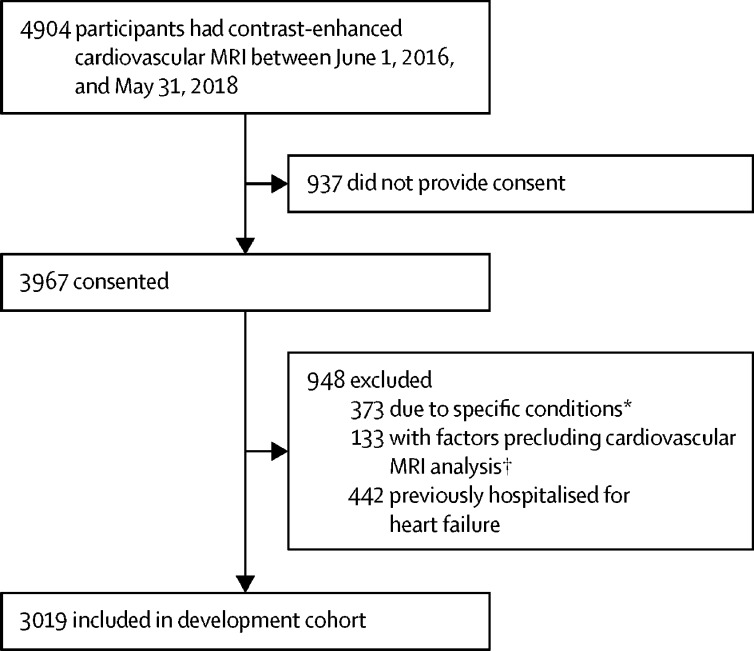
STROBE diagram STROBE=Strengthening the Reporting of Observational studies in Epidemiology. *Specific conditions were acute myocarditis (n=15), amyloidosis (n=20), complex congenital heart disease (n=12), Fabry disease (n=39), hypertrophic cardiomyopathy (n=266), iron overload (n=7), and Takotsubo cardiomyopathy (n=14). †Factors precluding cardiovascular MRI analysis were abandoned scanning (n=59), artifacts (n=14), and incomplete scan availability (n=60).

Between June 1, 2016, and May 31, 2018, consecutive adult patients (aged ≥16 years) undergoing clinically indicated CMR at Manchester University National Health Service (NHS) Foundation Trust, Manchester, UK, were prospectively recruited into a development cohort (registered at ClinicalTrials.gov, NCT02326324; figure 1). Exclusion criteria included previous hospitalisation for heart failure, determined from review of medical notes supplemented by patient self-reporting, or a diagnosis of any of the following: amyloidosis, complex congenital heart disease, Fabry disease, hypertrophic cardiomyopathy, iron overload, myocarditis, and stress-induced cardiomyopathy. Patients were also excluded if their CMR scan was not suitable for analysis. This study was approved by North West-Greater Manchester West Research Ethics Committee and all participants provided written informed consent.

### Procedures

Data were managed by use of Research Electronic Data Capture (known as REDCap).^5^ Baseline characteristics were identified from medical records. CMR was done by use of two scanners (1·5 T Avanto and 3 T Skyra; Siemens Medical Imaging; Erlangen, Germany). Scanning comprised steady-state free precession cine imaging in standard long axis and short axis planes, basal and mid left ventricle short axis T1 mapping (MOdified Look-Locker Inversion Recovery) imaging before and after administration of a gadolinium-based contrast agent (gadoterate meglumine [Dotarem]; Guerbet; Paris, France), and late gadolinium enhancement imaging. CMR analysis was done by use of cvi42 (version 5.6.7; Circle Cardiovascular Imaging; Calgary, AB, Canada). Measurements of ventricular mass, volumetrics, ejection fraction, and atrial area were done in accordance with recommendations from the Society for Cardiovascular Magnetic Resonance.^6^ Global longitudinal strain was measured as described previously.^7^ We determined the presence of myocardial infarction using late gadolinium enhancement. The presence of non-infarct (atypical) late gadolinium enhancement was also recorded. Myocardial fibrosis was measured by use of the extracellular volume technique, according to recommendations from the Society for Cardiovascular Magnetic Resonance.^8^ N-terminal pro-B-type natriuretic peptide (NT-pro-BNP) and high-sensitivity cardiac troponin T were measured in the laboratory by the research team from blood sampling done on the day of the CMR (cobas e 411 immunoanalyser; Roche Diagnostics; Mannheim, Germany). All baseline data collection, including the CMR analysis, was done before receiving, and was therefore blinded to, the outcome data.

### Outcome

Our study outcome was a composite of admission to hospital (hospitalisation) for heart failure or all-cause mortality occurring after CMR. Time to the composite endpoint was defined as the time to the first event, with censoring at the last follow-up date if no event occurred. Outcome data were obtained from NHS Digital. Hospital Episode Statistics for Admitted Patient Care records were used to identify episodes of hospital admission and mortality status was derived from Hospital Episode Statistics-Office of National Statistics (civil registration) data. Hospital Episode Statistics data contain diagnostic coding from the International Classification of Diseases, 10th revision (ICD-10), to denote up to 20 diagnoses per episode of hospital admission or cause of death. Hospitalisation for heart failure was defined as the first hospitalisation following CMR in which heart failure (ICD-10 I50) was the primary diagnosis.^9^, ^10^, ^11^ All-cause mortality was classified as all instances of death after CMR. The follow-up period was from the beginning of recruitment (June 1, 2016) until Aug 19, 2020. NHS Digital provided the outcome data blinded to participant characteristics.

### Model development

Model development is reported in accordance with Transparent Reporting of a Multivariable Prediction Model for Individual Prognosis or Diagnosis guidelines.^12^ Candidate predictor variables were selected according to clinical practice and literature review. Where potential candidate variables provided similar clinical information and were intuitively highly correlated (eg, ventricular volumes and ejection fraction, and left ventricle wall thickness and mass), the variable considered to be the most informative according to expert clinical consensus was selected. In total, 30 candidate predictor variables (28 unique variables and two transformed variables) were considered: age; sex; race; Index of Multiple Deprivation; body-mass index; percutaneous coronary intervention; coronary artery bypass graft; stroke or transient ischaemic attack; peripheral vascular disease; diabetes; hypertension; raised cholesterol; chronic obstructive pulmonary disease (COPD); atrial fibrillation; past or current smoker; QRS complex duration; estimated glomerular filtration rate; NT-pro-BNP; In(NT-pro-BNP); high-sensitivity cardiac troponin T; ln(high-sensitivity cardiac troponin T); MRI field strength; left ventricular ejection fraction; indexed myocardial mass; global longitudinal strain; right ventricular ejection fraction; body surface area-indexed left atrial area; myocardial infarction; atypical late gadolinium enhancement; and myocardial extracellular volume.

We developed our model in a cohort of 3019 patients (the development cohort; figure 1). Having missing data for the candidate variables was rare (2443 [2·7%] of 90 570 values) but there was a high proportion of participants with incomplete data (1224 [40·5%] of 3019; appendix p 6). Missing data were unintentional and their absence was due to incomplete medical records, incomplete CMR, or blood sampling not being done; thus, data were assumed to be missing at random. Multiple imputation by chained equations was used to create 20 imputed datasets.^13^, ^14^, ^15^ Missing data were imputed from the candidate predictor and outcome variables by use of predictive mean matching. Imputation by use of the Nelson–Aalen estimator is presented in the appendix (p 43) for comparison.

Analysis used Cox proportional hazards modelling on a time-since-study-entry timescale. Model parameters were pooled according to current guidelines.^16^ Correlations between candidate predictors were assessed with Pearson's *r*. Correlations larger than 0·7 were considered to be strong and indicative of potential collinearity. We assessed continuous variables showing significant univariable associations with the outcome for linearity using fractional polynomial transformations in multivariable models.^17^ In the presence of multiply imputed data, a composite stepwise selection procedure was used to identify the parsimonious multivariable model.^13^ Stepwise model selection according to Akaike's Information Criterion was done separately in each of the imputed datasets. Variables present in more than 50% of models were then considered for inclusion in the parsimonious model by use of a multivariable model Wald statistic test. Schöenfeld residuals were used to evaluate the proportional hazards assumption and data were pooled with the D_2_ method.^13^, ^16^, ^18^

### Internal validation

The developed model was validated internally. Non-parametric bootstrapping was used to estimate optimism and examine model stability. In each of the 20 imputed datasets, the entire modelling process, including predictor variable selection, was repeated in 1000 bootstrap samples. Model performance measures were combined across imputed datasets according to current guidelines.^16^ Model performance was evaluated in terms of discrimination and calibration. Discrimination indicates how well the model separates individuals who do and who do not develop the outcome. Discrimination was measured by use of Harrell's C-index, for which 0·5 indicates no discrimination beyond chance and 1·0 indicates perfect discrimination. Calibration assesses the agreement between predicted and observed risk, and was evaluated graphically at 3 years (1095 days) by use of a spline plot of the predicted versus observed probability of event-free survival. Each predicted survival probability estimated by the selected model was compared with the observed survival probability estimated by hazard regression across the bootstrap resamples, and a smoothed curve was overlaid.^18^, ^19^, ^20^ The Integrated Calibration Index, which is the mean difference between predicted and observed probabilities weighted to the distribution of predicted outcome probabilities, and E_90_, the 90th percentile of the absolute difference between observed and predicted probabilities, were calculated.^20^

### External validation

To assess model transportability, the final model was validated in an external validation cohort (appendix p 15). Between June 1, 2010, and March 25, 2016, consecutive adult patients undergoing clinically indicated CMR at the University of Pittsburgh Medical Center Cardiovascular Magnetic Resonance Center, Pittsburgh, PA, USA, were prospectively recruited. Exclusion criteria were identical to those for the development cohort. The follow-up period was from the beginning of recruitment (June 1, 2010) until April 18, 2017. The research protocol in the validation cohort was reviewed and approved by the University of Pittsburgh Institutional Review Board and all participants provided written informed consent.

A high proportion of participants (1242 [87·8%] of 1414) in the validation cohort had complete data; therefore, complete cases were used for the analysis. Some candidate predictor variables were not available in the validation dataset (Index of Multiple Deprivation, peripheral vascular disease, QRS complex duration, high-sensitivity cardiac troponin T, body surface area-indexed left atrial area, and right ventricular ejection fraction). To account for these missing variables, a reduced version of the final model was developed in the development cohort. Specifically, we re-conducted the entire model development and internal validation processes, while excluding the predictors that were unavailable in the validation cohort. NT-pro-BNP was also not available in the validation dataset; however, BNP was available in the validation cohort, and, given that they provide similar information, BNP was substituted for NT-pro-BNP in the model.

Using the pooled, optimism-adjusted calibration slope as a uniform shrinkage factor, all the predictor effects in the final developed model were penalised to account for overfitting before carrying out external validation.^21^ In the external validation cohort, discrimination was also assessed by use of Harrell's C-index, and calibration was assessed graphically at 1 year and 3 years by use of a scatter plot of predicted versus observed probability of event-free survival by decile, estimated by a Kaplan–Meier survival function.^22^ Recalibration via baseline hazard updating was done to account for the different risk profile of the validation cohort.^21^ Kaplan–Meier curves for predicted risk groups by quartile were plotted for the development and validation cohorts to provide further assessment of model discrimination and calibration. All analyses were done by use of R (version 4.0.3). In a preliminary analysis, we compared model discrimination when heart failure was the primary diagnosis versus when heart failure was the primary or secondary diagnosis.

### Model specification

A risk calculator that provides an estimated risk of hospitalisation for heart failure or all-cause mortality at 3 years for individual patients was generated from the externally validated optimism-adjusted model. The probability of hospitalisation for heart failure or all-cause mortality was estimated by use of the following equation, derived from the Cox proportional hazards model: *P*=1 – *S*_0_(t)^exp(prognostic index)^ where *S*_0_(t) is the baseline survival probability at time *t* (eg, 3 years) and the prognostic index is the sum of the products of the coefficients and the associated predictor.

### Role of the funding source

The funders of the study had no role in study design, data collection, data analysis, data interpretation, or writing of the report. Guerbet Laboratories and Roche Diagnostics International conducted a factual accuracy check of this manuscript, but any decisions to incorporate comments were made solely at the discretion of the authors.

## Results

The baseline characteristics of the development cohort (n=3019) are summarised in table 1. The median follow-up duration was 1118 days (IQR 950–1324). The composite outcome of hospitalisation for heart failure or all-cause mortality occurred in 225 (7·5%) of 3019 patients (annualised outcome rate 2·4% per year). 95 patients were hospitalised for heart failure and 152 patients died. 128 events occurred in patients with complete data and 97 events occurred in patients with one or more missing predictors. Outcome data were available for all patients. No patients were lost to follow-up or withdrew from the study.

**Table 1:** Baseline characteristics of the development cohort

		**Development cohort (n=3019)**	**Proportion missing**
**Demographics**			
Age, years		58 (46–68)	0
Sex			
	Female	1112 (36·8%)	0
	Male	1907 (63·2%)	0
Race			0
	White	2517 (83·4%)	..
	Asian	133 (4·4%)	..
	Black	81 (2·7%)	..
	Other	48 (1·6%)	..
	Not declared	240 (7·9%)	..
Index of Multiple Deprivation			36 (1·2%)
	1–10% (most deprived)	430/2983 (14·4%)	..
	11–20%	281/2983 (9·4%)	..
	21–30%	262/2983 (8·8%)	..
	31–40%	295/2983 (9·9%)	..
	41–50%	236/2983 (7·9%)	..
	51–60%	235/2983 (7·9%)	..
	61–70%	237/2983 (7·9%)	..
	71–80%	350/2983 (11·7%)	..
	81–90%	321/2983 (10·8%)	..
	91–100% (least deprived)	336/2983 (11·3%)	..
Body-mass index, kg/m^2^		27·8 (24·5–31·7)	48 (1·6%)
New York Heart Association class			117 (3·9%)
	No limitation	1083/2902 (37·3%)	..
	I	656/2902 (22·6%)	..
	II	615/2902 (21·2%)	..
	III	393/2902 (13·5%)	..
	IV	155/2902 (5·3%)	..
Heart failure stage*			0
	0	136 (4·5%)	..
	A	822 (27·2%)	..
	B	713 (23·6%)	..
	C	1348 (44·7%)	..
	D	0	..
Hospitalisation status at time of CMR (outpatient)		2859 (94·7%)	0
Referring centre			11 (0·4%)
Cardiac centre		1291/3008 (42·9%)	..
District hospitals		1717/3008 (57·1%)	..
**Medical history**			
Percutaneous coronary intervention		399 (13·2%)	0
Coronary artery bypass graft		181 (6·0%)	0
Stroke or transient ischaemic attack		206 (6·8%)	0
Peripheral vascular disease		117 (3·9%)	0
Diabetes		429 (14·2%)	0
Hypertension		1337 (44·3%)	0
Raised cholesterol		1302 (43·1%)	0
Chronic obstructive pulmonary disease		180 (6·0%)	0
Atrial fibrillation		417 (13·8%)	0
Current or past smoker		1465 (48·5%)	0
**Electrocardiogram and laboratory indices**			
Heart rate, beats per min		67·2 (12·6)	0
Sinus rhythm		2374/2609 (91·0%)	410 (13·6%)
Left bundle branch block		218/2605 (8·4%)	414 (13·7%)
Right bundle branch block		117/2597 (4·5%)	422 (14·0%)
QRS complex duration, ms		98 (90–109)	423 (14·0%)
Estimated glomerular filtration rate, mL/min		83 (70–90)	33 (1·1%)
N-terminal pro-B-type natriuretic peptide, pg/mL		127·1 (53·9–412·3)	675 (22·4%)
High-sensitivity cardiac troponin T, pg/mL		9·2 (4·5–15·3)	682 (22·6%)
**Cardiac structure and function**			
Left ventricular ejection fraction, %		56·2 (12·2)	3 (0·1%)
Indexed left ventricular end diastolic volume, mL/m^2^		89·7 (26·7)	13 (0·4%)
Indexed left ventricular end systolic volume, mL/m^2^		41·3 (24·4)	13 (0·4%)
Indexed left ventricle mass, g/m^2^		58·3 (17·7)	13 (0·4%)
Maximum left ventricle wall thickness, mm		10·6 (2·3)	16 (0·5%)
Left ventricle global longitudinal strain, %		−17·66 (4·48)	99 (3·3%)
Right ventricle ejection fraction, %		56·5 (9·4)	6 (0·2%)
Indexed right ventricle end diastolic volume, mL/m^2^		85·7 (23·1)	16 (0·5%)
Indexed right ventricle end systolic volume, mL/m^2^		38·2 (16·8)	16 (0·5%)
Indexed left atrial area, cm^2^/m^2^		14·15 (3·52)	30 (1·0%)
Infarct late gadolinium enhancement		663 (22·0%)	0
Atypical (non-infarct) late gadolinium enhancement		475 (15·7%)	0
Myocardial extracellular volume, %		26·1 (3·3)	381 (12·6%)

Data are median (IQR), n (%), n/N (%), or mean (SD). CMR=cardiovascular magnetic resonance.

* American College of Cardiology Foundation/American Heart Association staging system.

Univariable associations between candidate predictor variables and the composite outcome, pooled across 20 imputed datasets according to Rubin's rules, are presented in table 2. Global longitudinal strain and left ventricular ejection fraction were strongly correlated (*r*=–0·77), suggesting potential collinearity (appendix p 44). Both variables are generally considered to be important measurements of left ventricle function; therefore, we derived separate multivariable models that considered either global longitudinal strain or left ventricular ejection fraction. As global longitudinal strain was associated with a higher univariable Wald statistic than was left ventricular ejection fraction (table 2), we present the model considering global longitudinal strain in the main manuscript and the model considering left ventricular ejection fraction in the appendix (pp 3–4).

**Table 2:** Pooled univariable Cox regression for time to hospitalisation for heart failure or all-cause mortality in the development cohort

	**Hazard ratio (95% CI)**	**Wald χ^2^**	**p value**
Age	1·054 (1·042–1·065)	92·215	<0·0001
Male	0·681 (0·510–0·910)	6·814	0·0097
White race	1·321 (0·893–1·956)	1·960	0·16
Index of Multiple Deprivation	1·000 (1·000–1·000)	3·467	0·064
Body-mass index	1·016 (0·996–1·037)	2·421	0·12
Percutaneous coronary intervention	1·145 (0·790–1·660)	0·517	0·47
Coronary artery bypass graft	1·672 (1·064–2·626)	5·029	0·026
Stroke or transient ischaemic attack	1·848 (1·233–2·769)	8·955	0·0031
Peripheral vascular disease	2·844 (1·827–4·426)	21·675	<0·0001
Diabetes	2·353 (1·751–3·162)	32·542	<0·0001
Hypertension	1·295 (0·996–1·684)	3·754	0·054
Raised cholesterol	1·466 (1·127–1·908)	8·221	0·0045
Chronic obstructive pulmonary disease	3·414 (2·404–4·848)	47·604	<0·0001
Atrial fibrillation	1·670 (1·209–2·307)	9·783	0·0020
Past or current smoker	1·650 (1·261–2·158)	13·483	0·0003
QRS complex duration	1·007 (1·003–1·011)	14·597	0·0002
Estimated glomerular filtration rate	0·975 (0·966–0·984)	30·598	<0·0001
N-terminal pro-B-type natriuretic peptide	1·000 (1·000–1·000)	98·924	<0·0001
Ln(N-terminal pro-B-type natriuretic peptide)	1·856 (1·688–2·040)	166·548	<0·0001
High-sensitivity cardiac troponin T	1·000 (1·000–1·001)	1·810	0·18
Ln(high-sensitivity cardiac troponin T)	1·719 (1·514–1·952)	73·149	<0·0001
MRI field strength	1·121 (0·862–1·459)	0·734	0·39
Left ventricular ejection fraction	0·943 (0·935–0·952)	167·393	<0·0001
Indexed myocardial mass	1·021 (1·015–1·026)	52·616	<0·0001
Global longitudinal strain	1·197 (1·166–1·229)	177·882	<0·0001
Right ventricular ejection fraction	0·956 (0·944–0·968)	53·251	<0·0001
Indexed left atrial area	1·081 (1·052–1·110)	33·030	<0·0001
Myocardial infarction (infarct late gadolinium enhancement)	3·400 (2·613–4·424)	83·928	<0·0001
Atypical late gadolinium enhancement	1·536 (1·117–2·112)	7·053	0·0085
Myocardial extracellular volume	1·179 (1·140–1·218)	95·272	<0·0001

Models considering fractional polynomial transformations of continuous covariates showed that linear transformations were most frequently selected across the 20 imputed datasets, except for NT-pro-BNP, which was most frequently fit with natural logarithmic transformation (appendix p 7), in keeping with previous studies.^23^ The parsimonious multivariable model comprised age, diabetes, COPD, ln(NT-pro-BNP), global longitudinal strain, right ventricular ejection fraction, body surface area-indexed left atrial area, myocardial infarction (infarct late gadolinium enhancement), and myocardial extracellular volume (appendix pp 8–9).

Variable selection was consistent across imputed datasets. QRS complex duration was present as an additional variable in the model in 11 of the 20 datasets; however, nested multivariable Wald tests pooled across the 20 datasets showed that QRS complex duration did not significantly contribute to model performance (p=0·17).

The multivariable model satisfied the proportional hazards assumption. Univariably, global longitudinal strain did show some evidence of an association with time (appendix p 10); however, plots of the scaled Schöenfeld residuals against time in the 20 imputed datasets showed that this relationship was negligible (appendix p 11). Alternative models allowing penalised spline fits for continuous covariates satisfied the proportional hazards assumption at univariable and multivariable levels (appendix p 12), but model improvement was negligible.^24^ In light of these results, and to facilitate future clinical utility of the model, the linear expression of global longitudinal strain was preserved.

The median optimism-adjusted C-index for the parsimonious model across the 20 imputed datasets was 0·806 (95% CI 0·789–0·828; appendix p 13). The 3-year calibration plot showing the predictive accuracy of the model is presented in the appendix (p 14). As is observed from the plot, model calibration was high across the full range of predicted risk, with the calibration curve lying on or close to the reference line throughout. The median calibration slope (0·929; appendix p 13), the Integrated Calibration Index (0·001), and E_90_ (0·002) all indicated excellent calibration.

The external validation cohort comprised 1242 patients (appendix p 15). Their baseline characteristics are summarised in the appendix (p 16). Compared with the development cohort, patients in the external validation cohort were of similar age and ethnicity, a similar proportion were male versus female, and mean left ventricular ejection fraction was similar (table 1; appendix p 16). In the validation cohort, diabetes was more common and median body-mass index was higher, compared with the development cohort. The median follow-up duration in the validation cohort was 2117 days (IQR 1685–2446). The composite outcome of hospitalisation for heart failure or all-cause mortality occurred in 219 (17·6%) of 1242 patients (annualised outcome rate 3·0% per year). 70 patients were hospitalised for heart failure and 149 patients died. Outcome data were available for all patients. No patients were lost to follow-up or withdrew from the study.

The entire model building process, including variable selection, was repeated in the external validation cohort, excluding candidate predictors that were not available in the validation cohort. Variable selection in this further model derivation was consistent between the 20 imputed datasets and also between the models derived from the full list of candidate predictor variables and the reduced list of predictors available in both cohorts. Indeed, the only differences in model construction were that body surface area-indexed left atrial area and right ventricular ejection fraction, which were not available in the validation cohort, were not in the model.

The updated parsimonious multivariable model for use during external validation comprised age, diabetes, COPD, ln(NT-pro-BNP), global longitudinal strain, myocardial infarction, and myocardial extracellular volume. Optimism-adjusted model results are presented in table 3 and unadjusted model results are presented in the appendix (p 17).

**Table 3:** Final optimism-adjusted pooled model coefficients for the updated parsimonious multivariable model for use during external validation for time to hospitalisation for heart failure or all-cause mortality

	**Hazard ratio (95% CI)**	**Wald χ^2^**	**p value**
Age	1·026 (1·014–1·037)	19·344	<0·0001
Diabetes	1·437 (1·078–1·917)	6·171	0·014
Chronic obstructive pulmonary disease	1·742 (1·239–2·449)	10·296	0·0015
Ln(N-terminal pro-B-type natriuretic peptide)	1·275 (1·118–1·455)	13·455	0·0004
Global longitudinal strain	1·073 (1·038–1·109)	17·526	<0·0001
Myocardial infarction	1·560 (1·196–2·036)	10·851	0·0012
Myocardial extracellular volume	1·083 (1·044–1·122)	18·844	<0·0001

Schöenfeld residual testing showed that the proportional hazards assumption was met, except, as before, for global longitudinal strain, which univariably showed an association with time (appendix p 18). However, once again, further investigation showed that this association was minimal (appendix p 19) and that alternative model fitting led to negligible model improvement (appendix p 20); thus, linear expression was preserved.

**Figure 2:**
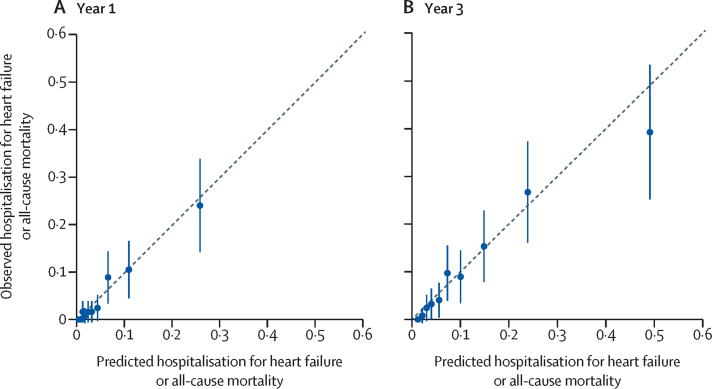
Recalibration plots for the optimism-adjusted multivariable model in the external validation cohort at 1 year and 3 years (A) Year 1. (B) Year 3. Risk groups are deciles.

**Figure 3:**
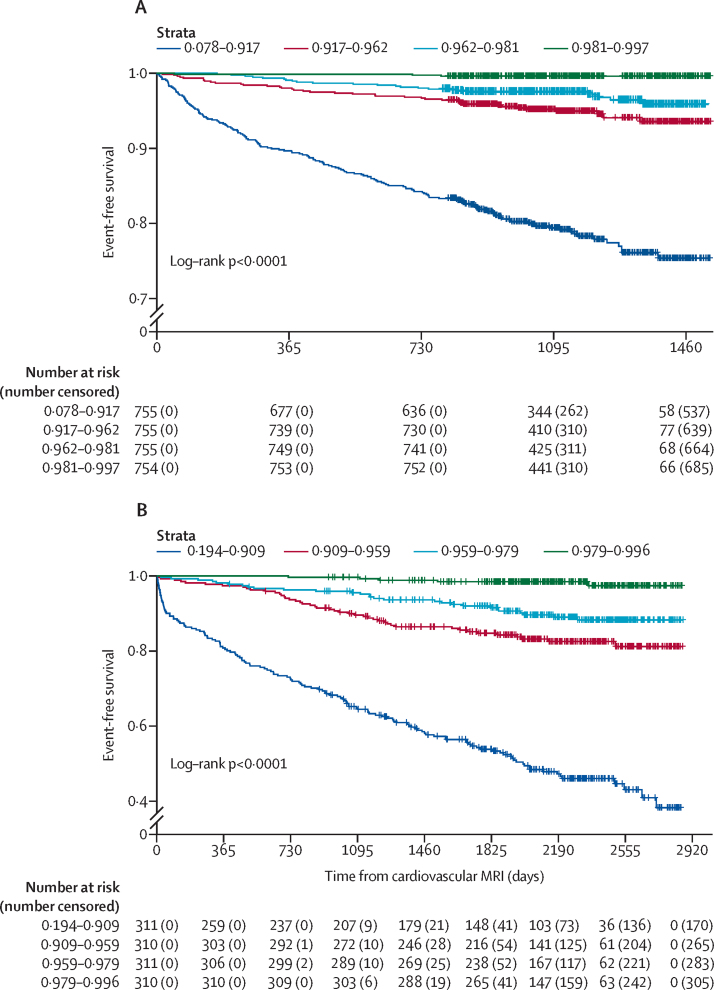
Kaplan–Meier curves of event-free survival Survival free of hospitalisation for heart failure and all-cause mortality in the model development (A) and external validation (B) cohorts, according to predicted probability. Cohorts were divided into quartiles according to predicted probability.

The median optimism-adjusted C-index for the externally validated model across the 20 imputed model development datasets was 0·805 (95% CI 0·793–0·829) in the development cohort, and the median calibration slope, adjusted for model-fitting optimism, was 0·943 (appendix p 21). The optimism-adjusted C-index for the model in the validation cohort was 0·793 (95% CI 0·766–0·820). The 1-year and 3-year optimism-adjusted calibration plots, updated to account for the different risk profile of the validation cohort, show that the model achieved good calibration (figure 2; calibration plots before baseline hazard updating are presented in the appendix [pp 22–23]). Kaplan–Meier curves also show good model performance, with clear separation of risk groups that is very similar for both cohorts (figure 3). A preliminary analysis comparing model discrimination when heart failure was the primary diagnosis versus when heart failure was the primary or secondary diagnosis showed similar performance (appendix p 42).

Based on the recalibrated model, the risk of hospitalisation for heart failure or all-cause mortality at 3 years for an individual patient can be calculated from the following equation: *Risk*=1 – 0·934^exp(prognostic index)^ where

*prognostic index*=(0·02524519 × age [in years]) + (0·36274393 × diabetes) + (0·55492737 × COPD) + (0·24307998 × ln[NT-pro-BNP in pg/mL]) + (0·07038957 × global longitudinal strain [in %]) + (0·44487534 × myocardial infarction) + (0·07935639 × myocardial extracellular volume [in %]),

after centring. The presence or absence of diabetes, COPD, and myocardial infarction is included as 1 or 0, respectively.

## Discussion

We present an externally validated risk prediction model of hospitalisation for heart failure and all-cause mortality in patients with, or at risk of, heart failure, but who have not been previously hospitalised for heart failure. The model was derived from, and validated in, large cohorts of patients with contemporary, deep phenotyping, and provides accurate, individualised estimates of risk. To our knowledge, it is the first such model.

Heart failure is common, has a poor prognosis, and its associated health-care costs, particularly related to hospitalisation for heart failure, are high. As the global population ages and factors predisposing to heart failure become more prevalent, identifying individuals with a higher likelihood of being hospitalised for heart failure or dying is, increasingly, a priority. Individual (personalised) risk stratification could allow the direction of intensified therapy and closer follow-up to those at increased risk, with the aim of preventing, or postponing, hospitalisation for heart failure and death, while redirecting unnecessary intervention away from those at low risk (increasing health-care efficiency and reducing medicalisation) and facilitating research into preventive strategies.^25^, ^26^

It is with this priority in mind that numerous heart failure prognostic models have been developed; however, few models have been validated and those that have were typically derived and validated in cohorts with a high proportion of patients who have been previously hospitalised for heart failure.^4^ Although such models are important, to some extent, they become relevant too late in the patient pathway—when patients have a dismal prognosis, intuitively the underlying disease mechanisms might be less modifiable, and the large expenditure associated with hospitalisation for heart failure has already been incurred. Conversely, our study included patients at a relatively earlier stage, with more than 50% in stage A or B heart failure, and patients who had been previously hospitalised for heart failure were excluded.^27^ Reflecting this study population, the annualised outcome rate was low (2·4% per year). Risk prediction in this group is understudied, despite the large potential benefit that can be gained from early intervention.

Unlike many previous models,^4^ our model considered variables fundamental to contemporary heart failure management, including circulating biomarkers (eg, natriuretic peptides and high-sensitivity troponin) and electrocardiograph measurements (eg, QRS complex duration).^4^, ^28^, ^29^, ^30^ Furthermore, our model considered modern measurements of cardiac structure, tissue character, and tissue function, including late gadolinium enhancement (the gold-standard assessment for myocardial infarction),^31^ myocardial extracellular volume (a robust measurement of myocardial fibrosis),^8^ global longitudinal strain (a more sensitive measure of mechanical dysfunction than ejection fraction),^32^ and left atrial size.^33^ All of these variables have shown prognostic utility; however, none have been evaluated in the context of other contemporary heart failure biomarkers in large development and validatory cohorts. Compared with other imaging modalities like echocardiography, CMR also provides considerably more accurate and reliable measurement of standard measures, such as left ventricle mass and right ventricular ejection fraction, allowing more definitive assessment of their prognostic utility. We did not consider medications as candidate variables because they are not an underlying pathophysiological cause of risk, but instead have a physician-influenced downstream effect.

The final externally validated model is intuitive. It includes age, key comorbidities that are in keeping with previous studies (ie, diabetes and COPD),^4^ natriuretic peptide concentrations, and left ventricle function (global longitudinal strain or left ventricular ejection fraction; see appendix [p 32]) and structure (the presence of myocardial infarction and myocardial fibrosis). The number of variables in the parsimonious model is less than in many previous models,^4^, ^34^ and the linear expression of variables was used, both of which should facilitate clinical utilisation. The study population was not categorised according to left ventricular ejection fraction, the thresholds for which are arbitrary and variable,^2^ meaning that the model is more widely applicable and simpler. An aim of future work will be to produce an accessible calculator to facilitate clinical implementation.

Model performance was high; indeed, it substantially outperformed other validated heart failure prognostic models.^4^ This good performance appears to reflect the high prognostic value of myocardial fibrosis and global longitudinal strain, which, along with age, were the main contributors to the parsimonious model. Specifically, the derived model had good discriminative ability across both the development and external validation cohorts and was highly calibrated across the full risk profile.

Khan and colleagues^35^ have previously derived race-specific and sex-specific models for the 10-year risk of incident heart failure in the general population using age, blood pressure, fasting glucose, body-mass index, cholesterol, smoking status, and QRS complex duration. We considered an analogue of all these variables in our model building process (except for blood pressure, data for which were only available for approximately 40% of participants, precluding appropriate imputation), and, through variable selection procedures, showed that they were less informative than the variables that were included in the final validated model. Furthermore, our model is, to a large extent, based on disease processes underlying patients' risk of hospitalisation for heart failure or all-cause mortality, providing insight into potentially targetable mechanisms.

A limitation of this study is that all patients were undergoing CMR and so the population is potentially skewed. However, the CMR service at Manchester University NHS Foundation Trust serves several district hospitals across the northwest of England, in addition to Manchester University NHS Foundation Trust (the tertiary cardiac centre); indeed, 57·1% of patients in the development cohort were from district hospitals. Thus, our study population is more representative than those of many studies that have derived heart failure prognostic models, which have often been restricted to specialist centres, have used randomised controlled trial populations, or have recruited specific heart failure subgroups.^4^ It is only through recruiting patients who are undergoing clinical CMR that the prognostic value of contrast-enhanced CMR measurements in populations with cardiovascular disease can be evaluated at a meaningful scale. For comparison, although studies, such as the UK Biobank^36^ and the Multi-Ethnic Study of Atherosclerosis,^37^ have involved conducting CMR in large cohorts, all or almost all scans are done without contrast agent administration, and few patients have cardiovascular disease.

The recruitment period for the validation cohort preceded that for the development cohort. However, recruitment criteria were identical and image acquisition and analysis adhered to recommendations from the Society for Cardiovascular Magnetic Resonance. Cohorts with the required level of contemporary deep phenotyping to enable validation of the derived model are rare; indeed, as far as we are aware, the validation cohort is the only such cohort. External validation is a strength of our study, representing rigorous use of the available data. The high performance of our model in the external validation cohort, despite some differences in cohort characteristics compared with the development cohort, shows the robustness of the model.

Defining hospitalisation for heart failure as the first hospitalisation following CMR in which heart failure was the primary diagnosis is specific and in keeping with other studies,^38^ but could have resulted in admissions being missed in which heart failure was concomitant with other diagnoses. However, a preliminary analysis comparing model discrimination when heart failure was the primary diagnosis versus when heart failure was the primary or secondary diagnosis showed similar performance.

Another potential limitation of our model is its applicability, given that many patients do not have access to CMR. However, given that stratified and precision medicine are the focus of much of medical science, it is hoped that showing the unique prognostic value of CMR measurements of cardiac structure and function will catalyse clinical implementation.

In conclusion, we developed and externally validated a risk prediction model that utilises contemporary deep phenotyping to provide accurate, individualised estimates of risk for hospitalisation for heart failure and all-cause mortality in patients with, or at risk of, heart failure, but who have not previously been hospitalised for heart failure.

## Data sharing

Deidentified participant data will be made available to requesters 1 year after the date of publication, with no end date to availability. Data will be shared after an appropriate proposal is submitted and can be used for any purpose. Proposals should be directed to christopher.miller@manchester.ac.uk. Requesters will be required to sign a data access agreement.

## Declaration of interests

EBS serves as an adviser for HAYA Therapeutics and consults for PureTech Health. PFB was in receipt of a Joint Alliance Medical and University Hospital of South Manchester Fellowship Salary Support Grant. JHN has a part-time appointment at Bioxydyn. TM serves as the clinical lead for the National Heart Failure Audit and has received speaker fees from Novartis, AstraZeneca, and Vifor. CAM has served on advisory boards for Novartis, Boehringer Ingelheim and Lilly Alliance, and AstraZeneca; serves as an adviser for HAYA Therapeutics and PureTech Health; and has received research support from Amicus Therapeutics, Guerbet Laboratories, Roche, and Univar Solutions (none are relevant to the contents of this Article, except where described in the Role of the funding source). All other authors declare no competing interests.
