# Supplementary material for: Predicting hospitalisation for heart failure and death in patients with, or at risk of, heart failure before first hospitalisation: a retrospective model development and external validation study
Source: Lancet Digit Health. 2022 May 10;4(6):e445–54. doi: 10.1016/S2589-7500(22)00045-0 (PMC9130210; doi:10.1016/S2589-7500(22)00045-0)
Supplement: Supplementary appendix [file mmc1.pdf]

THE LANCET
Digital Health

Supplementary appendix

This appendix formed part of the original submission and has been peer reviewed. We post it as supplied by the authors.

Supplement to: Bradley J, Schelbert EB, Bonnett LJ, et al. Predicting hospitalisation for heart failure and death in patients with, or at risk of, heart failure before first hospitalisation: a retrospective model development and external validation study. *Lancet Digit Health* 2022; published online May 10. https://doi.org/10.1016/S2589-7500(22)00045-0.

Table of Contents

SUPPLEMENTAL RESULTS 3

PROGNOSTIC MODELLING THAT CONSIDERED LEFT VENTRICULAR EJECTION FRACTION, RATHER THAN GLOBAL LONGITUDINAL STRAIN, AS A CANDIDATE PREDICTOR 3

MODEL PERFORMANCE – INTERNAL VALIDATION 3
MODEL PERFORMANCE – EXTERNAL VALIDATION 3
INDIVIDUALISED RISK CALCULATOR 4

SUPPLEMENTAL TABLES AND FIGURES 5

SUPPLEMENTAL FIGURE 1. MISSING DATA PATTERN IN THE DEVELOPMENT COHORT 6
SUPPLEMENTAL TABLE 1. FREQUENCY OF OCCURRENCE OF POLYNOMIAL TRANSFORMATIONS IN THE DEVELOPMENT MULTIVARIABLE MODELS FROM THE 20 IMPUTED DATASETS 7
SUPPLEMENTAL TABLE 2A. OPTIMISM-ADJUSTED PARSIMONIOUS MULTIVARIABLE MODEL 8
SUPPLEMENTAL TABLE 2B. UNADJUSTED PARSIMONIOUS MULTIVARIABLE MODEL 9
SUPPLEMENTAL TABLE 3. UNIVARIABLE AND MODEL SCHÖENFELD TEST RESULTS FOR THE PARSIMONIOUS MODEL IN THE 20 IMPUTED DATASETS, POOLED WITH THE D_2 METHOD FOR χ^2 VALUES 10
SUPPLEMENTAL FIGURE 2. SCALED SCHÖENFELD RESIDUAL PLOTS FOR GLOBAL LONGITUDINAL STRAIN (GLS) AGAINST TIME IN THE 20 IMPUTED DATASETS 11
SUPPLEMENTAL TABLE 4. MODEL DISCRIMINATION FOR THE 'ORIGINAL' PARSIMONIOUS MODEL AND FOR AN ALTERNATIVE MODEL USING PENALISED SMOOTHING SPLINES FOR CONTINUOUS VARIABLES 12
SUPPLEMENTAL TABLE 5. INTERNAL VALIDATION ('MODEL PERFORMANCE') RESULTS FOR THE PARSIMONIOUS MULTIVARIABLE MODEL 13
SUPPLEMENTAL FIGURE 3. CALIBRATION PLOT OF BOOTSTRAP RESAMPLING ESTIMATES OF PREDICTED PROBABILITY OF HOSPITALISATION FOR HEART FAILURE OR ALL-CAUSE MORTALITY AT 3-YEARS VERSUS OBSERVED PROBABILITIES USING THE FLEXIBLE HAZARD REGRESSION APPROACH[5,6] 14
SUPPLEMENTAL FIGURE 4. STROBE DIAGRAM FOR THE EXTERNAL VALIDATION COHORT 15
SUPPLEMENTAL TABLE 6. BASELINE CHARACTERISTICS OF THE EXTERNAL VALIDATION COHORT 16
SUPPLEMENTAL TABLE 7. UNADJUSTED POOLED MODEL COEFFICIENTS FOR THE EXTERNALLY VALIDATED PARSIMONIOUS MULTIVARIABLE MODEL FOR TIME TO HOSPITALISATION FOR HEART FAILURE OR ALL-CAUSE MORTALITY 17
SUPPLEMENTAL TABLE 8. UNIVARIABLE AND MODEL SCHÖENFELD TEST RESULTS FOR THE PARSIMONIOUS EXTERNALLY VALIDATED MODEL IN THE 20 IMPUTED DATASETS, POOLED WITH THE D_2 METHOD FOR χ^2 VALUES 18
SUPPLEMENTAL FIGURE 5. SCALED SCHÖENFELD RESIDUAL PLOTS FOR GLOBAL LONGITUDINAL STRAIN (GLS) AGAINST TIME IN THE 20 IMPUTED DATASETS FOR THE EXTERNALLY VALIDATED MODEL 19
SUPPLEMENTAL TABLE 9. MODEL DISCRIMINATION FOR THE 'ORIGINAL' EXTERNALLY VALIDATED PARSIMONIOUS MODEL AND FOR AN ALTERNATIVE MODEL USING PENALISED SMOOTHING SPLINES FOR CONTINUOUS VARIABLES 20
SUPPLEMENTAL TABLE 10. INTERNAL VALIDATION ('MODEL PERFORMANCE') RESULTS FOR THE FINAL, EXTERNALLY VALIDATED PARSIMONIOUS MULTIVARIABLE MODEL 21
SUPPLEMENTAL FIGURE 6. CALIBRATION PLOTS PRIOR TO BASELINE HAZARD UPDATING FOR THE OPTIMISM-ADJUSTED MULTIVARIABLE MODEL IN THE EXTERNAL VALIDATION COHORT AT 1- AND 3- YEARS. 22
SUPPLEMENTAL FIGURE 7. CALIBRATION PLOTS PRIOR TO BASELINE HAZARD UPDATING FOR THE UNADJUSTED MULTIVARIABLE MODEL IN THE EXTERNAL VALIDATION COHORT AT 1- AND 3- YEARS. 23
SUPPLEMENTAL TABLE 11. FREQUENCY OF OCCURRENCE OF POLYNOMIAL TRANSFORMATIONS IN THE DEVELOPMENT MULTIVARIABLE MODELS FROM THE 20 IMPUTED DATASETS 24
SUPPLEMENTAL TABLE 12A. OPTIMISM-ADJUSTED PARSIMONIOUS MULTIVARIABLE MODEL 25
SUPPLEMENTAL TABLE 12B. UNADJUSTED PARSIMONIOUS MULTIVARIABLE MODEL 26
SUPPLEMENTAL TABLE 13. UNIVARIABLE AND MODEL SCHÖENFELD TEST RESULTS FOR THE PARSIMONIOUS MODEL IN 20 IMPUTED DATASETS, POOLED WITH THE D_2 METHOD FOR χ^2 VALUES 27
SUPPLEMENTAL FIGURE 8. SCHÖENFELD RESIDUAL PLOTS FOR LV EJECTION FRACTION AGAINST TIME IN THE 20 IMPUTED DATASETS 28
SUPPLEMENTAL TABLE 14. MODEL DISCRIMINATION FOR THE 'ORIGINAL' PARSIMONIOUS MODEL AND FOR AN ALTERNATIVE MODEL USING PENALISED SMOOTHING SPLINES FOR CONTINUOUS VARIABLES 29
SUPPLEMENTAL TABLE 15. INTERNAL VALIDATION ('MODEL PERFORMANCE') RESULTS FOR THE PARSIMONIOUS MULTIVARIABLE MODEL 30

SUPPLEMENTAL FIGURE 9. CALIBRATION PLOT OF BOOTSTRAP RESAMPLING ESTIMATES OF PREDICTED PROBABILITY OF HOSPITALISATION FOR HEART FAILURE OR ALL-CAUSE MORTALITY AT 3-YEARS VERSUS OBSERVED PROBABILITIES USING THE FLEXIBLE HAZARD REGRESSION APPROACH[5,6] 31
SUPPLEMENTAL TABLE 16. OPTIMISM-ADJUSTED POOLED MODEL COEFFICIENTS FOR THE EXTERNALLY VALIDATED PARSIMONIOUS MULTIVARIABLE MODEL FOR TIME TO HOSPITALISATION FOR HEART FAILURE OR ALL-CAUSE MORTALITY 32
SUPPLEMENTAL TABLE 17. UNADJUSTED POOLED MODEL COEFFICIENTS FOR THE EXTERNALLY VALIDATED PARSIMONIOUS MULTIVARIABLE MODEL FOR TIME TO HOSPITALISATION FOR HEART FAILURE OR ALL-CAUSE MORTALITY 33
SUPPLEMENTAL TABLE 18. UNIVARIABLE AND MODEL SCHÖENFELD TEST RESULTS FOR THE EXTERNALLY VALIDATED PARSIMONIOUS MODEL IN 20 IMPUTED DATASETS, POOLED WITH THE D_2 METHOD FOR χ^2 VALUES 34
SUPPLEMENTAL FIGURE 10. SCALED SCHÖENFELD RESIDUAL PLOTS FOR LV EJECTION FRACTION AGAINST TIME IN THE 20 IMPUTED DATASETS FOR THE EXTERNALLY VALIDATED MODEL 35
SUPPLEMENTAL TABLE 19. MODEL DISCRIMINATION FOR THE 'ORIGINAL' EXTERNALLY VALIDATED PARSIMONIOUS MODEL AND AN ALTERNATIVE MODEL USING PENALISED SMOOTHING SPLINES FOR CONTINUOUS COVARIABLES 36
SUPPLEMENTAL TABLE 20. INTERNAL VALIDATION ('MODEL PERFORMANCE') RESULTS FOR THE FINAL, EXTERNALLY VALIDATED PARSIMONIOUS MULTIVARIABLE MODEL 37
SUPPLEMENTAL FIGURE 11. RE-CALIBRATION PLOTS FOR THE OPTIMISM-ADJUSTED MULTIVARIABLE MODEL IN THE EXTERNAL VALIDATION COHORT AT 1- AND 3- YEARS. 38
SUPPLEMENTAL FIGURE 12. CALIBRATION PLOTS PRIOR TO BASELINE HAZARD UPDATING FOR THE OPTIMISM-ADJUSTED MULTIVARIABLE MODEL IN THE EXTERNAL VALIDATION COHORT AT 1- AND 3-YEARS. 39
SUPPLEMENTAL FIGURE 13. CALIBRATION PLOTS PRIOR TO BASELINE HAZARD UPDATING FOR THE UNADJUSTED MULTIVARIABLE MODEL IN THE EXTERNAL VALIDATION COHORT AT 1- AND 3- YEARS. 40
SUPPLEMENTAL FIGURE 14. SURVIVAL FREE OF HOSPITALISATION FOR HEART FAILURE AND ALL-CAUSE MORTALITY IN THE MODEL DEVELOPMENT (A) AND EXTERNAL VALIDATION (B) COHORTS, ACCORDING TO PREDICTED PROBABILITY. 41
SUPPLEMENTAL TABLE 21. PRELIMINARY ANALYSIS COMPARING MODEL DISCRIMINATION WHEN HEART FAILURE WAS THE PRIMARY DIAGNOSIS VERSUS WHEN HEART FAILURE WAS THE PRIMARY OR SECONDARY DIAGNOSIS 42
SUPPLEMENTAL TABLE 22. COMPARISON OF IMPUTED VARIABLE FOR IMPUTATION MODELS INCLUDING THE CANDIDATE PREDICTORS, OUTCOME STATUS VARIABLE AND TIME-TO-OUTCOME OR THE NELSON-AALEN ESTIMATE 43
SUPPLEMENTAL FIGURE 15. CORRELATIONS BETWEEN CANDIDATE PREDICTORS 44

FURTHER ANALYSES 45

MODEL PERFORMANCE BY HEART FAILURE STAGE 45

SUPPLEMENTAL TABLE 23. PRELIMINARY COMPARISON OF MODEL DISCRIMINATION FOR PATIENTS WITH STAGE A/B HEART FAILURE VERSUS STAGE C/D 45

REFERENCES 46

Supplemental results

Prognostic modelling that considered left ventricular ejection fraction, rather than global longitudinal strain, as a candidate predictor

Models considering fractional polynomial transformations of continuous covariates showed that linear transformations were most frequently selected across the 20 imputed datasets, excepting NT-proBNP, which was most frequently fit with natural logarithmic transformation (Supplemental Table 11), which is in keeping with previous studies.[1]

The parsimonious multivariable model included age, diabetes, COPD, Ln (NT-proBNP), LV ejection fraction, right ventricle ejection fraction, body surface area-indexed left atrial area, myocardial infarction and myocardial ECV (Supplemental Table 12A and B).

Model performance – internal validation

Variable selection was consistent across imputed datasets. QRS duration was present as an additional variable in the model in 18 of the 20 datasets, however nested multivariable Wald tests pooled across the 20 datasets showed that QRS duration did not significantly contribute to model performance ($p = 0{\cdot}073$).

The multivariable model satisfied the proportional hazards assumption, evaluated using Schöenfeld residual testing, pooled with the D_2 method.[2,3] Univariably, LV ejection fraction did demonstrate some evidence of an association with time (Supplemental Table 13), however plots of the scaled Schöenfeld residuals against time in the 20 imputed datasets demonstrated that this relationship was negligible (Supplemental Figure 8). Alternative models allowing penalised spline fits for continuous covariates satisfied the proportional hazards assumption at univariable and multivariable levels (Supplemental Table 14), however model improvement was negligible.[4] In light of this, and to facilitate future clinical utility of the model, the linear expression of global longitudinal strain was preserved.

The median optimism-adjusted C-index for the parsimonious model across the 20 imputed datasets was 0·810 (95% CI 0·796 - 0·833) (Supplemental Table 15). The 3-year calibration plot demonstrating the predictive accuracy of the model is presented in Supplemental Figure 9. As is observed from the plot, model calibration was high across the full range of predicted risk, with the calibration curve lying on or close to the reference line throughout. The calibration slope (0·928, Supplemental Table 15), ICI (0·002) and E_{90} (0·003) all indicated excellent calibration.

Model performance – external validation

The model was re-derived in the development cohort using the matched pool of candidate predictor variables. Variable selection was consistent across the 20 imputed datasets, and between the models derived from the original candidate predictor variables and the matched pool. Indeed, the only differences in model construction were that indexed left atrial area and right ventricular ejection fraction, which were not available in the validation cohort, were not in the model.

The final, externally validated, parsimonious multivariable model included: age, diabetes, COPD, Ln (NT-proBNP), LV ejection fraction, myocardial infarction and myocardial ECV. Optimism-adjusted model results are presented in Supplemental Table 16. (Unadjusted model results are presented in Supplemental Table 17).

Schöenfeld residual testing demonstrated that the proportional hazards assumption was met, except, as before, for LV ejection fraction, which univariably, again demonstrated an association with time (Supplemental Table 18). However, once again, further investigation demonstrated that this association was minimal (Supplemental Figure 10), and that alternative model fitting led to negligible model improvement (Supplemental Table 19), thus the linear expression was preserved.

The median optimism-adjusted C-index for the externally validated model across the 20 imputed model development datasets was 0·809 (95% CI 0·797 – 0·832), and the calibration slope, adjusted for model fitting optimism, was 0·943 (Supplemental Table 20). The optimism-adjusted C-index for the model in the validation cohort was 0·787 (95% CI 0·760 – 0·814). (Unadjusted discrimination was similar). The 1- and 3-year optimism-adjusted calibration plots, updated to account for the different risk profile of the validation cohort, demonstrate that the model achieves good calibration (Supplemental Figure 11; calibration plots prior to baseline hazard updating are presented in Supplemental Figures 12 and 13). Kaplan-Meier curves also demonstrate good model performance (Supplemental Figure 14).

Individualised risk calculator

The risk of HHF or all-cause mortality at three years for an individual patient can be calculated from the following equation:

$$Risk = 1 - 0 \cdot 930^{exp(Prognostic\ Index)}$$

where Prognostic Index = 0·02844767*age (years) + 0·39214302*diabetes + 0·75343827*COPD + 0·22732893*$ln(NT - proBNP)$ (pg/mL) – 0·02674360*LV ejection fraction (%) + 0·41937295*myocardial infarction + 0·07347436*myocardial ECV (%), after centring. Presence or absence of diabetes, COPD and myocardial infarction are included as a 1 or 0 respectively.

Supplemental tables and figures

Supplemental Figure 1. Missing data pattern in the development cohort

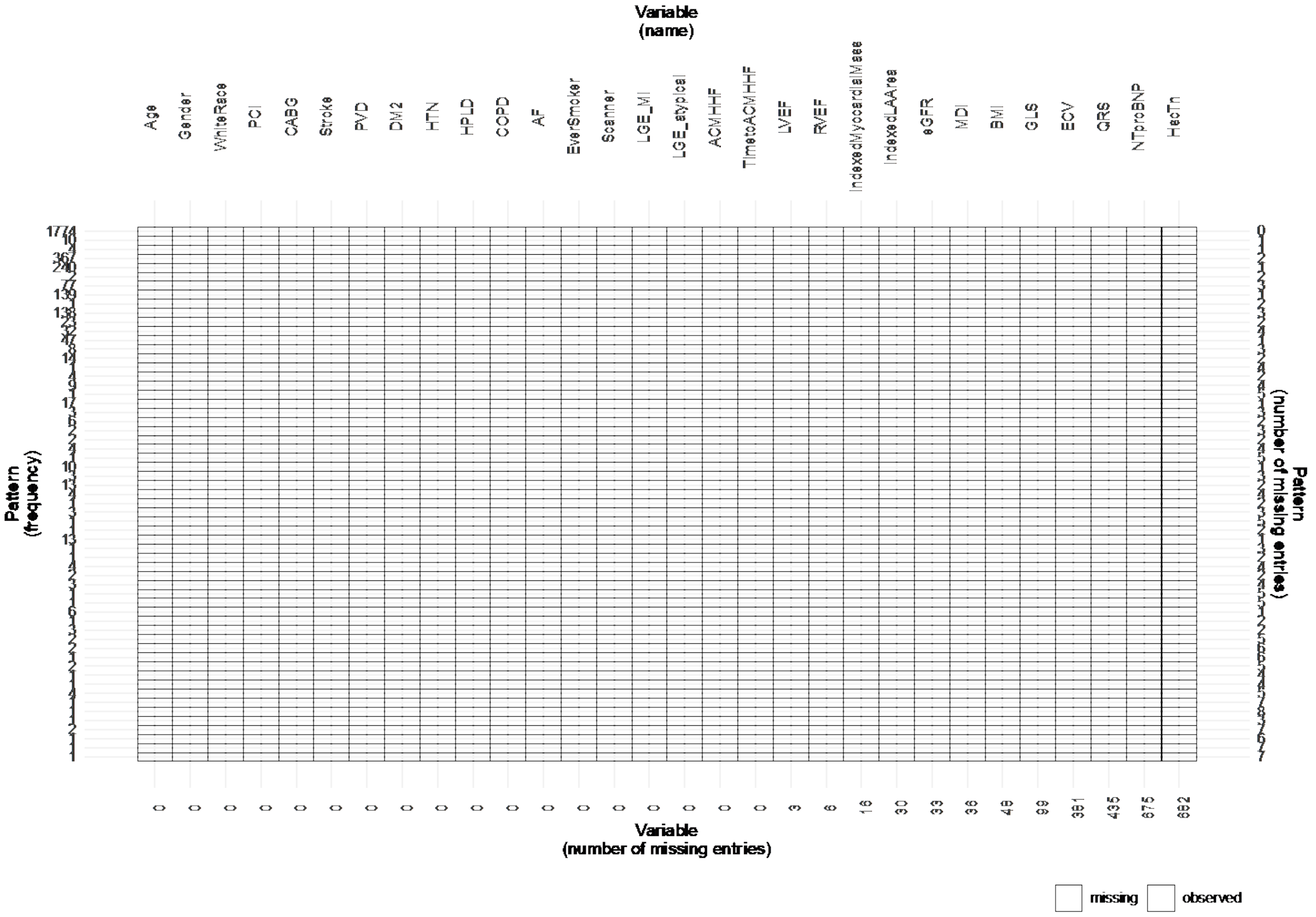

ACMHHF=hospitalisation for heart failure or all-cause mortality. AF=atrial fibrillation. BMI=body mass index. DM2=diabetes. eGFR=estimated glomerular filtration rate. HPLD=raised cholesterol. HTN=Hypertension. IndexedLAArea=indexed left atrial area. LGE_atypical=atypical late gadolinium enhancement. LGE_MI=myocardial infarction. LVEF=left ventricle ejection fraction. MDI=Index Multiple Deprivation. PVD=peripheral vascular disease. QRS=QRS complex duration. RVEF=right ventricle ejection fraction. Scanner=MRI field strength. TimetoACMHHF=Time to first hospitalisation for heart failure or all-cause mortality. Other abbreviations per previous tables.

Supplemental Table 1. Frequency of occurrence of polynomial transformations in the development multivariable models from the 20 imputed datasets

Transformation of covariable	Frequency
Age / 100	20
COPD	20
Diabetes	20
ECV / 10	20
(GLS + 30·4) /10	10
Indexed left atrial area / 10	11
Infarct LGE	20
Ln (NT-proBNP / 1000)	19
$(\text{NT-proBNP} / 1000)^{0\cdot5}$	1
NT-proBNP / 1000	1
RVEF / 100	12

Abbreviations per previous tables and figures.

Supplemental Table 2A. Optimism-adjusted parsimonious multivariable model

	**Hazard Ratio**	**95% Confidence Interval**	**p value**
Age	1·030	1·018 - 1·042	<0·0001
Diabetes	1·408	1·055 - 1·879	0·020
COPD	1·669	1·185 - 2·351	0·0035
Ln (NT-proBNP)	1·332	1·160 - 1·530	0·0001
Indexed left atrial area	0·951	0·917 - 0·987	0·0084
GLS	1·044	1·007 - 1·083	0·021
RVEF	0·981	0·968 - 0·994	0·0044
Infarct LGE	1·596	1·223 - 2·082	0·0006
ECV	1·094	1·054 - 1·136	<0·0001

Abbreviations per previous tables.

Supplemental Table 2B. Unadjusted parsimonious multivariable model

	Hazard Ratio	95% Confidence Interval	Wald χ^2	p value
Age	1·032	1·020 - 1·045	26·170	<0·0001
Diabetes	1·445	1·059 - 1·972	5·457	0·020
COPD	1·736	1·201 - 2·508	8·709	0·0035
Ln (NT-proBNP)	1·362	1·173 - 1·580	16·912	<0·0001
Indexed left atrial area	0·948	0·910 - 0·986	7·103	0·0084
GLS	1·047	1·007 - 1·089	5·415	0·021
RVEF	0·979	0·965 - 0·993	8·302	0·0044
Infarct LGE	1·653	1·242 - 2·201	12·004	0·0006
ECV	1·101	1·058 - 1·147	22·469	<0·0001

Abbreviations per previous tables.

Supplemental Table 3. Univariable and model Schöenfeld test results for the parsimonious model in the 20 imputed datasets, pooled with the D_2 method for χ^2 values

	Imputed dataset																				
	**1**	**2**	**3**	**4**	**5**	**6**	**7**	**8**	**9**	**10**	**11**	**12**	**13**	**14**	**15**	**16**	**17**	**18**	**19**	**20**	**Pooled**
Age	0·62	0·64	0·63	0·60	0·64	0·62	0·61	0·62	0·60	0·57	0·67	0·62	0·57	0·61	0·61	0·60	0·61	0·57	0·56	0·64	0·61
Diabetes	0·51	0·54	0·50	0·47	0·55	0·53	0·56	0·49	0·51	0·47	0·48	0·49	0·48	0·54	0·50	0·57	0·56	0·52	0·52	0·50	0·52
COPD	0·46	0·49	0·47	0·50	0·46	0·49	0·44	0·46	0·46	0·49	0·46	0·49	0·50	0·50	0·49	0·51	0·47	0·50	0·49	0·48	0·48
Ln (NT-proBNP)	0·0088	0·016	0·058	0·13	0·035	0·017	0·33	0·05	0·09	0·075	0·0094	0·07	0·086	0·12	0·086	0·041	0·022	0·10	0·06	0·0091	0·074
Indexed left atrial area	0·90	0·99	0·93	0·96	0·96	0·79	0·98	0·90	0·99	1·00	0·99	0·77	0·78	0·92	0·90	0·72	0·97	0·77	0·91	0·89	0·91
GLS	0·0091	0·010	0·0096	0·0084	0·011	0·0075	0·017	0·012	0·012	0·012	0·0086	0·013	0·0083	0·013	0·018	0·0083	0·0072	0·014	0·0074	0·0076	0·011
RVEF	0·40	0·35	0·40	0·43	0·43	0·38	0·45	0·42	0·47	0·42	0·38	0·39	0·39	0·42	0·40	0·39	0·39	0·45	0·40	0·40	0·41
Infarct LGE	0·16	0·18	0·15	0·18	0·18	0·16	0·17	0·18	0·20	0·19	0·17	0·18	0·19	0·18	0·18	0·17	0·17	0·18	0·19	0·16	0·18
ECV	0·57	0·12	0·50	0·82	0·41	0·33	0·44	0·29	0·70	0·32	0·49	0·27	0·93	0·75	0·80	0·43	0·54	0·85	0·85	0·13	0·55
**Multivariable model**	0·06	0·08	0·18	0·18	0·17	0·10	0·33	0·17	0·22	0·21	0·06	0·24	0·20	0·29	0·28	0·18	0·10	0·25	0·15	0·040	0·25

Abbreviations per previous tables.

Supplemental Figure 2. Scaled Schöenfeld residual plots for global longitudinal strain (GLS) against time in the 20 imputed datasets

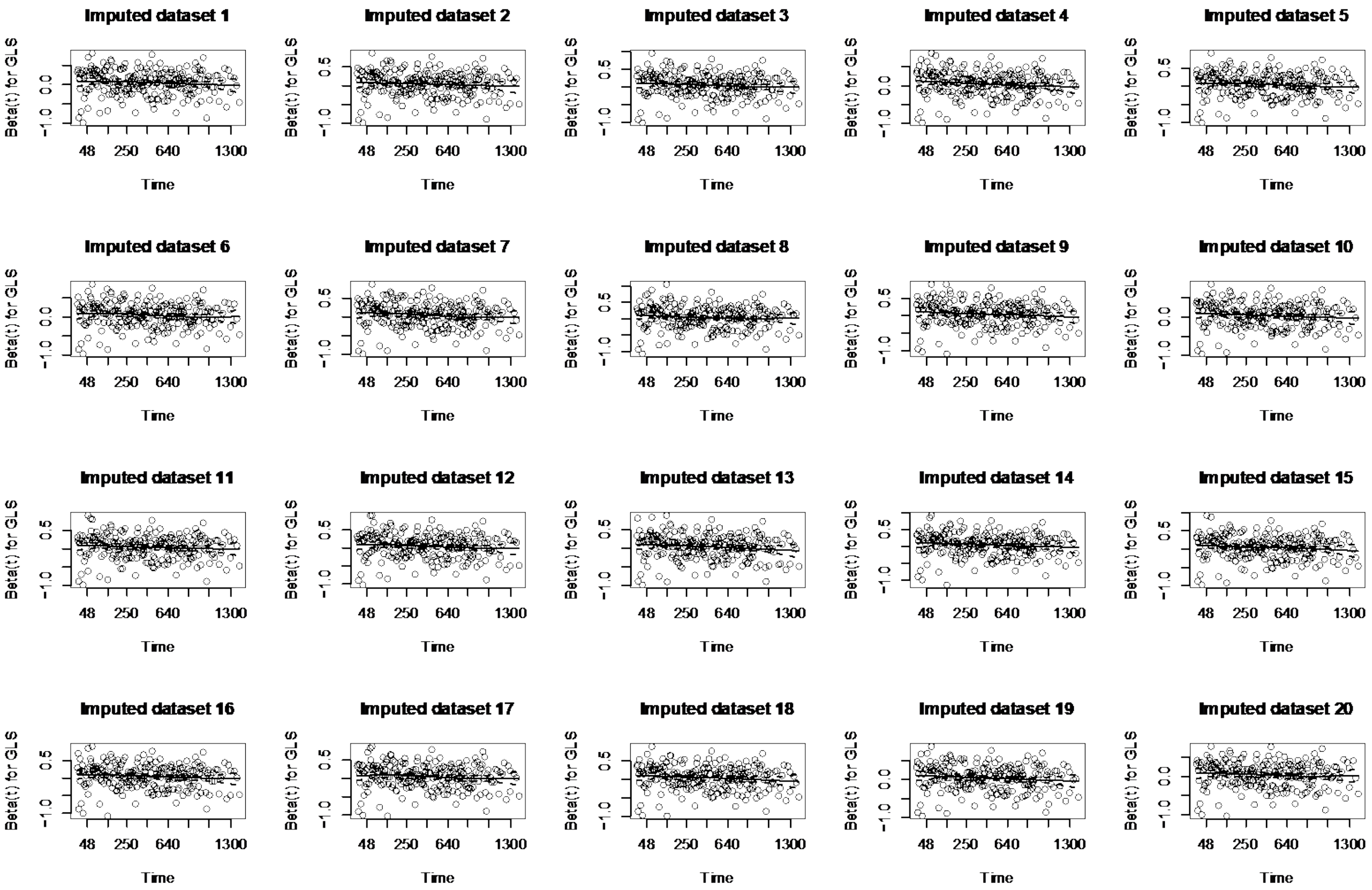

Supplemental Table 4. Model discrimination for the ‘original’ parsimonious model and for an alternative model using penalised smoothing splines for continuous variables

	**Model**	
**Imputed dataset**	**Original**	**pspline**
1	0·814	0·825
2	0·817	0·828
3	0·815	0·825
4	0·819	0·828
5	0·816	0·826
6	0·821	0·828
7	0·816	0·824
8	0·818	0·828
9	0·815	0·825
10	0·819	0·828
11	0·816	0·827
12	0·817	0·828
13	0·815	0·825
14	0·818	0·830
15	0·815	0·827
16	0·816	0·826
17	0·818	0·829
18	0·817	0·828
19	0·820	0·831
20	0·822	0·832
**Median**	0·817	0·828

pspline=penalised smoothing spline.

Supplemental Table 5. Internal validation ('model performance') results for the parsimonious multivariable model

**Imputed dataset**	**Discrimination**	**Optimism adjusted C-index**	**Slope**
1	0·814	0·803	0·930
2	0·817	0·806	0·929
3	0·815	0·802	0·925
4	0·819	0·808	0·932
5	0·816	0·804	0·930
6	0·821	0·809	0·927
7	0·816	0·804	0·930
8	0·818	0·806	0·927
9	0·816	0·803	0·929
10	0·819	0·808	0·933
11	0·816	0·803	0·929
12	0·818	0·806	0·926
13	0·815	0·804	0·933
14	0·819	0·807	0·929
15	0·815	0·803	0·934
16	0·816	0·804	0·924
17	0·818	0·806	0·925
18	0·817	0·806	0·929
19	0·820	0·808	0·933
20	0·822	0·811	0·929
**Median**	0·817	0·806	0·929

Supplemental Figure 3. Calibration plot of bootstrap resampling estimates of predicted probability of hospitalisation for heart failure or all-cause mortality at 3-years versus observed probabilities using the flexible hazard regression approach[5,6]

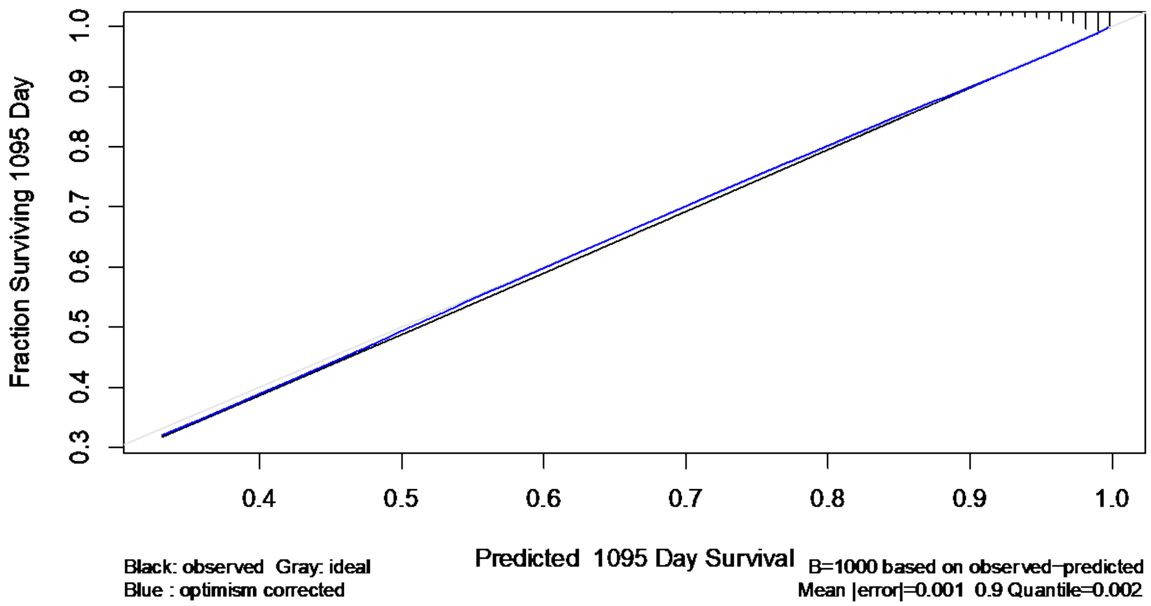

The smooth black line is the apparent calibration, and the blue line is the bootstrap optimism- (overfitting-) corrected calibration curve, both estimated by adaptive linear spline hazard regression. The grey line is the line of identity and represents perfect calibration. Mean |error| is equivalent to the ICI and 0.9 quantile is equivalent to E_{90}.[7] A rug plot of the distribution of predicted outcome probabilities sits on the top axis of the plot. Survival is survival free of hospitalisation for heart failure or all-cause mortality.

Supplemental Figure 4. STROBE diagram for the external validation cohort

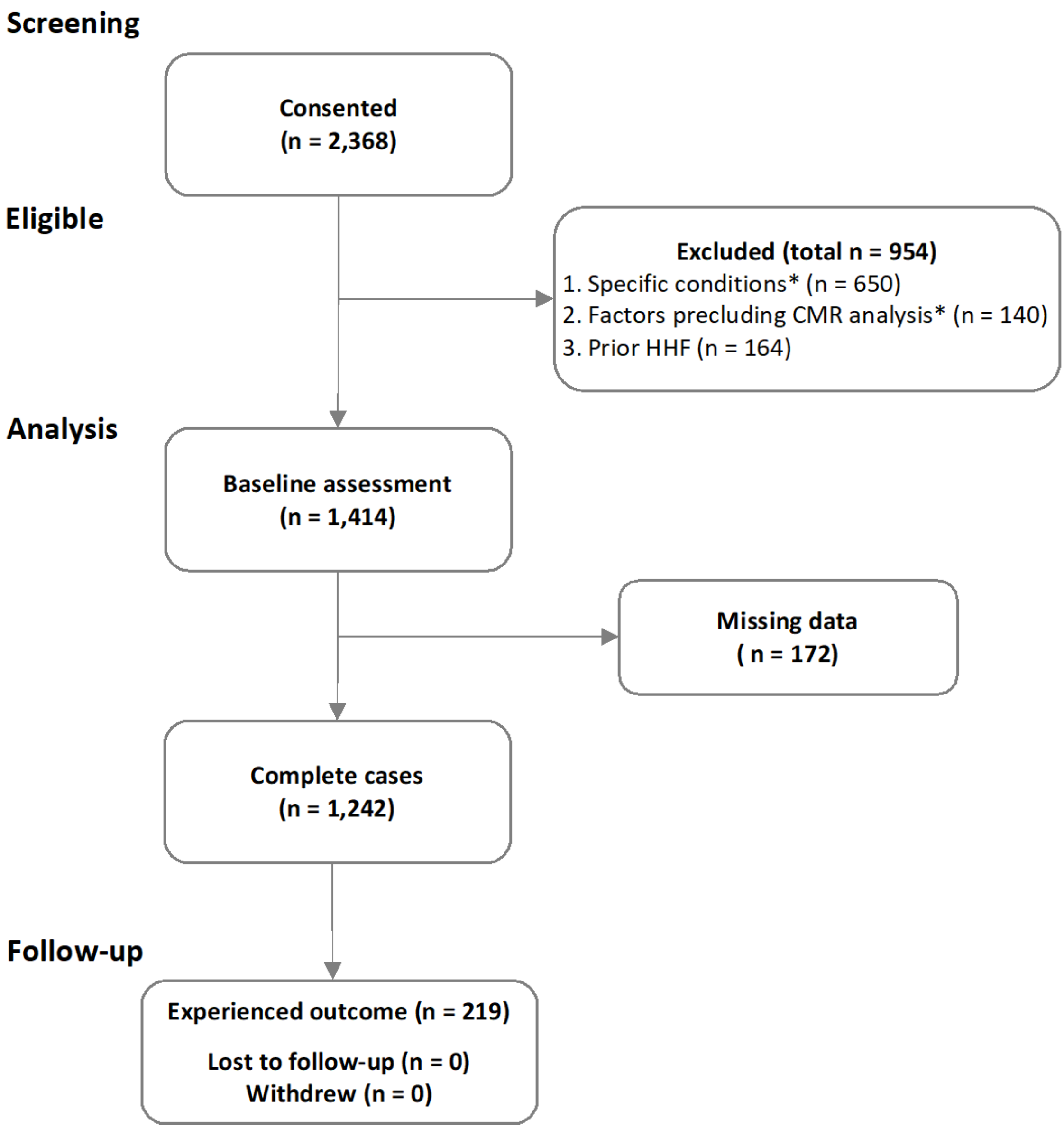

*Specific conditions: amyloidosis (n = 68), complex congenital heart disease (n = 339), Fabry disease (n = 3), hypertrophic cardiomyopathy (n = 221), iron-overload (n = 5), Stress cardiomyopathy (n = 14). Factors precluding CMR analysis: inadequate image quality to permit retrospective electrocardiography-gated segmented breath-held cine acquisition (n = 140). CMR=cardiovascular magnetic resonance. HHF=hospitalisation for heart failure.

Supplemental Table 6. Baseline characteristics of the external validation cohort

	**n=1,242**
Age, years	56 [44-65]
Male	702 (56·5%)
White Race	1100 (88·6%)
Index of Multiple Deprivation	N/A
Body mass index, kg/m^2	28·2 [24·4 – 33·2]
Percutaneous coronary intervention	146 (11·8%)
Coronary artery bypass graft	88 (7·1%)
Stroke or transient ischaemic attack	50 (4·0%)
Peripheral vascular disease	N/A
Diabetes	223 (18·0%)
Hypertension	592 (47·7%)
Raised Cholesterol	461 (37·1%)
Chronic obstructive pulmonary disease	47 (3·8%)
Atrial fibrillation	113 (9·1%)
Current or past smoker	517 (41·6%)
QRS complex duration, ms	N/A
Estimated glomerular filtration rate, mL/min	90 [73-100]
B-type natriuretic peptide	63·5 [26 – 184]
High-sensitivity troponin-T	N/A
MRI field strength (1·5T)	1242 (100%)
Left ventricle ejection fraction, %	54·7 ± 14·4
Indexed left ventricular mass, g/m^2	58·8 ± 20·1
Global longitudinal strain, %	-15·7 ± 4·8
Right ventricle ejection fraction, %	N/A
Indexed left atrial area, cm^2/m^2	N/A
Infarct late gadolinium enhancement	246 (19·8%)
Atypical (non-infarct) late gadolinium enhancement	205 (16·5%)
Myocardial extracellular volume, %	27·9 ± 3·9

Data are median (IQR), n (%), or mean (SD)

Supplemental Table 7. Unadjusted pooled model coefficients for the externally validated parsimonious multivariable model for time to hospitalisation for heart failure or all-cause mortality

	Hazard Ratio	95% Confidence Interval	Wald χ^2	p value
Age	1·027	1·015 - 1·040	19·344	<0·0001
Diabetes	1·469	1·083 - 1·994	6·171	0·014
COPD	1·802	1·255 - 2·587	10·296	0·0015
Ln (NT-proBNP)	1·294	1·125 - 1·488	13·455	0·0004
GLS	1·078	1·040 - 1·116	17·526	<0·0001
Infarct LGE	1·603	1·209 - 2·126	10·851	0·0012
ECV	1·088	1·047 - 1·130	18·844	<0·0001

Abbreviations per previous tables.

Supplemental Table 8. Univariable and model Schöenfeld test results for the parsimonious externally validated model in the 20 imputed datasets, pooled with the D_2 method for χ^2 values

	**Imputed dataset**																				
	**1**	**2**	**3**	**4**	**5**	**6**	**7**	**8**	**9**	**10**	**11**	**12**	**13**	**14**	**15**	**16**	**17**	**18**	**19**	**20**	**Pooled**
Age	0·60	0·52	0·60	0·58	0·62	0·60	0·58	0·60	0·58	0·55	0·64	0·60	0·56	0·59	0·59	0·58	0·59	0·55	0·55	0·62	0·59
Diabetes	0·55	0·59	0·53	0·52	0·58	0·57	0·58	0·52	0·54	0·52	0·52	0·52	0·52	0·57	0·54	0·58	0·58	0·56	0·56	0·55	0·55
COPD	0·48	0·51	0·48	0·51	0·48	0·50	0·46	0·47	0·47	0·50	0·48	0·50	0·51	0·51	0·50	0·51	0·48	0·51	0·52	0·50	0·49
Ln (NT-proBNP)	0·0076	0·015	0·056	0·12	0·029	0·16	0·28	0·045	0·075	0·062	0·0079	0·060	0·068	0·10	0·076	0·038	0·021	0·081	0·049	0·0080	0·064
GLS	0·0060	0·0070	0·0061	0·0052	0·0065	0·0052	0·0095	0·0075	0·0072	0·0075	0·0053	0·0080	0·0047	0·0084	0·011	0·0054	0·0044	0·0083	0·0044	0·0049	0·0066
Infarct LGE	0·16	0·18	0·16	0·18	0·17	0·16	0·17	0·18	0·19	0·19	0·17	0·17	0·19	0·18	0·17	0·17	0·17	0·18	0·19	0·16	0·17
ECV	0·55	0·12	0·49	0·85	0·38	0·33	0·40	0·28	0·63	0·30	0·48	0·25	0·99	0·74	0·75	0·42	0·54	0·78	0·82	0·14	0·53
**Multivariable model**	0·051	0·081	0·11	0·10	0·11	0·072	0·17	0·13	0·13	0·13	0·051	0·15	0·10	0·16	0·16	0·10	0·069	0·14	0·083	0·054	0·12

Abbreviations per previous tables.

Supplemental Figure 5. Scaled Schöenfeld residual plots for global longitudinal strain (GLS) against time in the 20 imputed datasets for the externally validated model

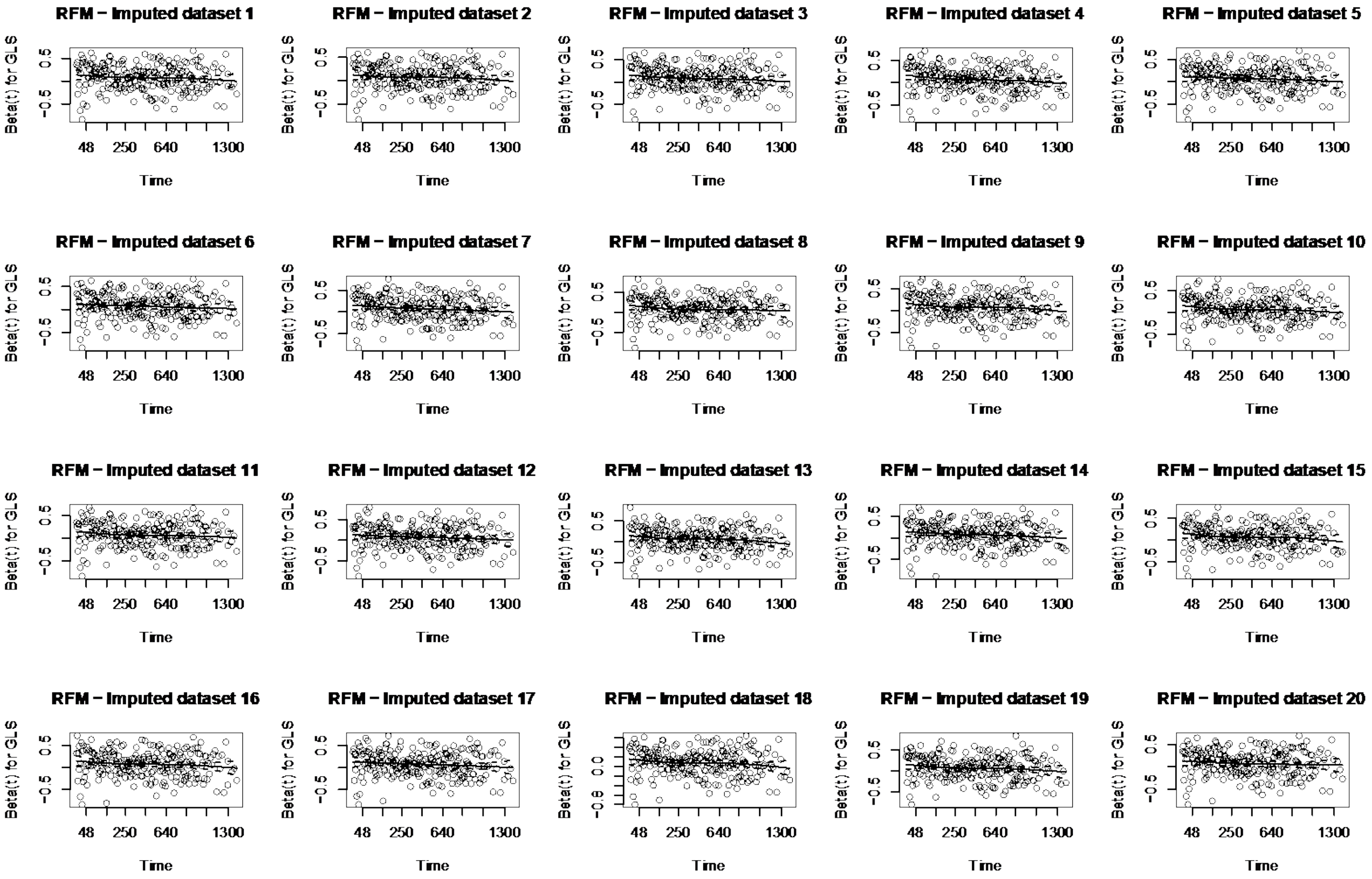

Supplemental Table 9. Model discrimination for the 'original' externally validated parsimonious model and for an alternative model using penalised smoothing splines for continuous variables

	Model	
**Imputed dataset**	**Original**	**pspline**
1	0·812	0·819
2	0·814	0·821
3	0·813	0·820
4	0·816	0·822
5	0·814	0·820
6	0·818	0·822
7	0·814	0·819
8	0·815	0·820
9	0·814	0·821
10	0·817	0·822
11	0·813	0·820
12	0·815	0·822
13	0·814	0·820
14	0·816	0·824
15	0·813	0·821
16	0·814	0·820
17	0·816	0·823
18	0·816	0·822
19	0·818	0·824
20	0·819	0·825
**Median**	0·815	0·821

Abbreviations per previous tables.

Supplemental Table 10. Internal validation ('model performance') results for the final, externally validated parsimonious multivariable model

Imputed dataset	Discrimination	Optimism adjusted C-index	Slope
1	0·812	0·802	0·944
2	0·814	0·805	0·943
3	0·813	0·803	0·938
4	0·816	0·807	0·943
5	0·814	0·804	0·942
6	0·818	0·809	0·944
7	0·814	0·805	0·940
8	0·815	0·806	0·942
9	0·814	0·804	0·940
10	0·817	0·807	0·944
11	0·813	0·802	0·940
12	0·815	0·805	0·942
13	0·814	0·804	0·945
14	0·816	0·807	0·940
15	0·813	0·804	0·948
16	0·814	0·804	0·938
17	0·816	0·806	0·938
18	0·816	0·806	0·944
19	0·818	0·808	0·943
20	0·819	0·810	0·943
**Median**	0·815	0·805	0·943

Supplemental Figure 6. Calibration plots prior to baseline hazard updating for the optimism-adjusted multivariable model in the external validation cohort at 1- and 3- years.

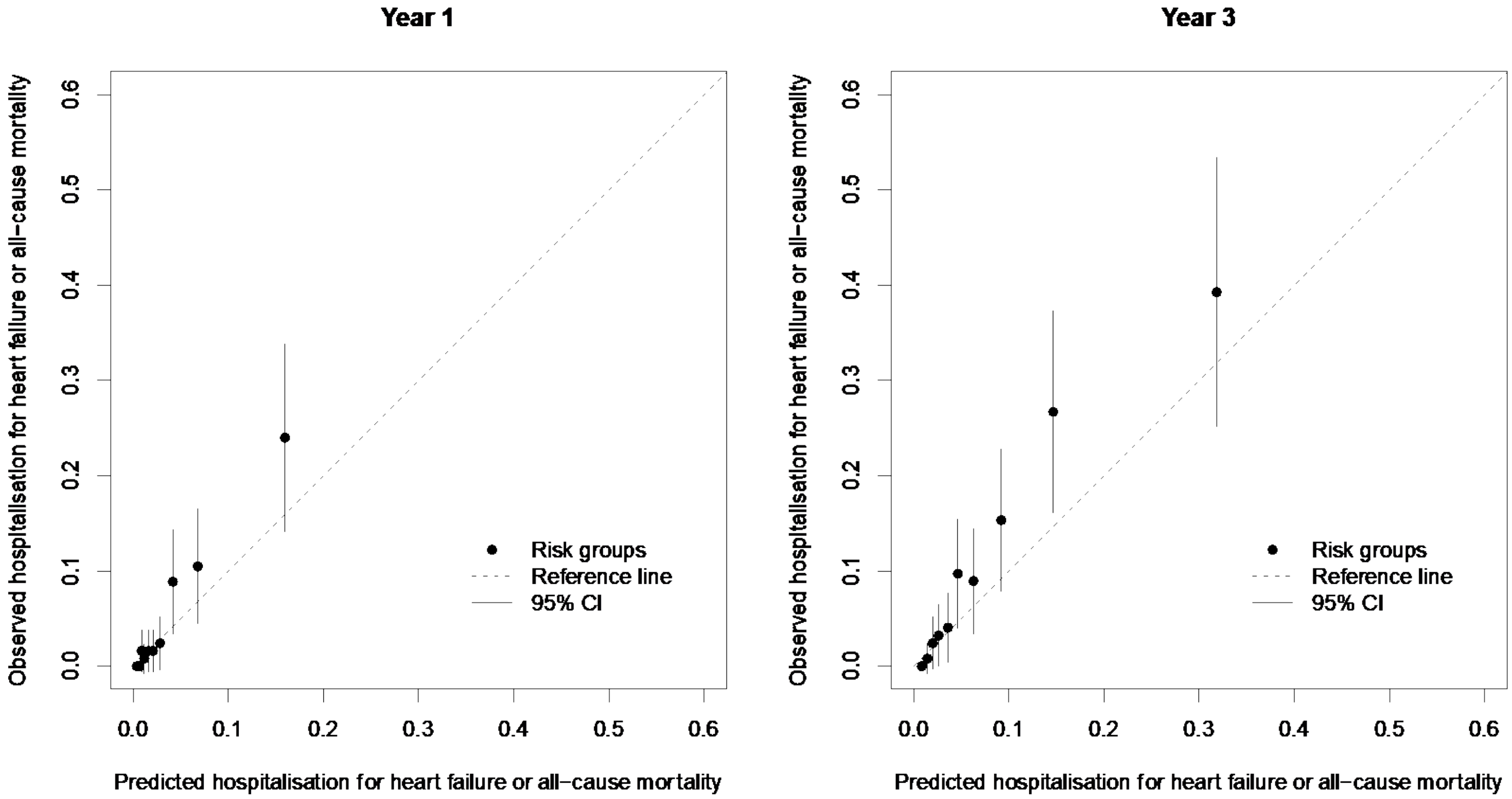

Supplemental Figure 7. Calibration plots prior to baseline hazard updating for the unadjusted multivariable model in the external validation cohort at 1- and 3-years.

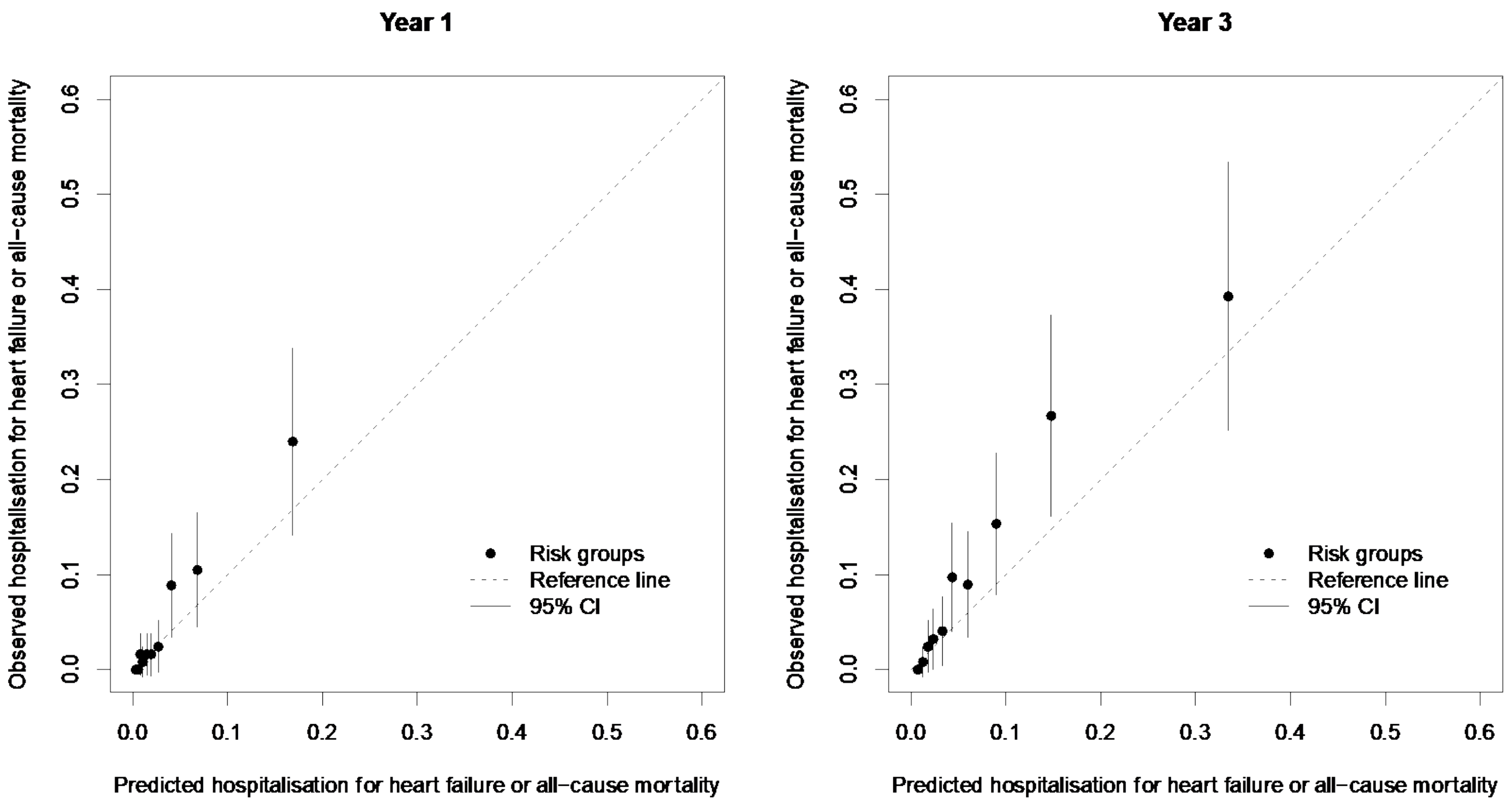

Supplemental Table 11. Frequency of occurrence of polynomial transformations in the development multivariable models from the 20 imputed datasets

**Transformation of covariable**	**Frequency**
Age / 100	20
COPD	20
Diabetes	20
ECV / 10	20
Indexed left atrial area / 10	6
Infarct LGE	20
$(LVEF / 100)^{-1}$	1
$(LVEF / 100)^{-2}$ * Ln (LVEF / 100)	1
$(LVEF / 100)^{-2}$	2
LVEF / 100	18
Ln (NT-proBNP / 1000)	19
RVEF / 100	2

Abbreviations per previous tables·

Supplemental Table 12A. Optimism-adjusted parsimonious multivariable model

	Hazard Ratio	95% Confidence Interval	p value
Age	1·031	1·020 - 1·043	<0·0001
Diabetes	1·427	1·070 - 1·905	0·016
COPD	1·897	1·346 - 2·672	0·0003
Ln (NT-proBNP)	1·315	1·147 - 1·509	0·0001
Indexed left atrial area	0·952	0·917 - 0·988	0·0091
LVEF	0·981	0·969 - 0·993	0·0019
RVEF	0·985	0·971 - 0·998	0·026
Infarct LGE	1·548	1·186 - 2·019	0·0014
ECV	1·089	1·048 - 1·131	<0·0001

Abbreviations per previous tables.

Supplemental Table 12B. Unadjusted parsimonious multivariable model

	**Hazard Ratio**	**95% confidence interval**	**Wald χ^2**	**p value**
Age	1·034	1·021 - 1·047	28·705	<0·0001
Diabetes	1·467	1·075 - 2·002	5·915	0·016
COPD	1·993	1·378 - 2·883	13·558	0·0003
Ln (NT-proBNP)	1·344	1·159 - 1·557	15·778	0·0001
Indexed left atrial area	0·948	0·911 - 0·987	6·942	0·0091
LVEF	0·980	0·967 - 0·992	9·914	0·0019
RVEF	0·984	0·969 - 0·998	5·013	0·026
Infarct LGE	1·601	1·202 - 2·132	10·473	0·0014
ECV	1·096	1·052 - 1·142	19·655	<0·0001

Abbreviations per previous tables.

Supplemental Table 13. Univariable and model Schöenfeld test results for the parsimonious model in 20 imputed datasets, pooled with the D₂ method for χ^2 values

	**Imputed dataset**																				
	**1**	**2**	**3**	**4**	**5**	**6**	**7**	**8**	**9**	**10**	**11**	**12**	**13**	**14**	**15**	**16**	**17**	**18**	**19**	**20**	**Pooled**
Age	0·62	0·63	0·62	0·60	0·62	0·60	0·60	0·61	0·59	0·56	0·65	0·56	0·56	0·60	0·60	0·59	0·60	0·55	0·55	0·63	0·60
Diabetes	0·48	0·51	0·47	0·44	0·52	0·49	0.52	0·46	0·47	0·44	0·45	0·46	0·45	0·52	0·47	0·53	0·53	0·48	0·48	0·48	0·48
COPD	0·45	0·48	0·47	0·49	0·45	0·49	0·44	0·45	0·46	0·48	0·45	0·48	0·49	0·50	0·49	0·50	0·46	0·49	0·48	0·47	0·47
Ln (NT-proBNP)	0·0086	0·016	0·055	0·13	0·035	0·016	0·32	0·052	0·089	0·078	0·0093	0·069	0·083	0·11	0·086	0·040	0·022	0·093	0·059	0·0090	0·072
Indexed left atrial area	0·88	0·97	0·90	0·95	0·94	0·77	1·00	0·92	0·98	0·99	0·97	0·76	0·76	0·90	0·90	0·69	0·96	0·75	0·89	0·91	0·90
LVEF	0·0060	0·0057	0·0052	0·0071	0·0068	0·0053	0·0060	0·0066	0·0071	0·0090	0·0055	0·0067	0·0060	0·0062	0·0072	0·0056	0·0054	0·0089	0·0059	0·0060	0·0064
RVEF	0·36	0·33	0·37	0·41	0·39	0·35	0·42	0·39	0·44	0·40	0·35	0·37	0·36	0·39	0·38	0·36	0·36	0·42	0·36	0·37	0·38
Infarct LGE	0·16	0·18	0·16	0·19	0·18	0·16	0·17	0·19	0·21	0·20	0·17	0·18	0·20	0·18	0·19	0·18	0·18	0·19	0·19	0·17	0·18
ECV	0·54	0·11	0·47	0·83	0·40	0·32	0·42	0·28	0·67	0·32	0·48	0·26	0·95	0·73	0·79	0·40	0·53	0·84	0·81	0·12	0·53
**Multivariable model**	0·07	0·08	017	0·22	0·18	0·10	0·27	0·17	0·22	0·24	0·068	0·24	0·2	0·26	0·23	0·19	0·12	0·26	0·18	0·047	0·16

Abbreviations per previous tables.

Supplemental Figure 8. Schöenfeld residual plots for LV ejection fraction against time in the 20 imputed datasets

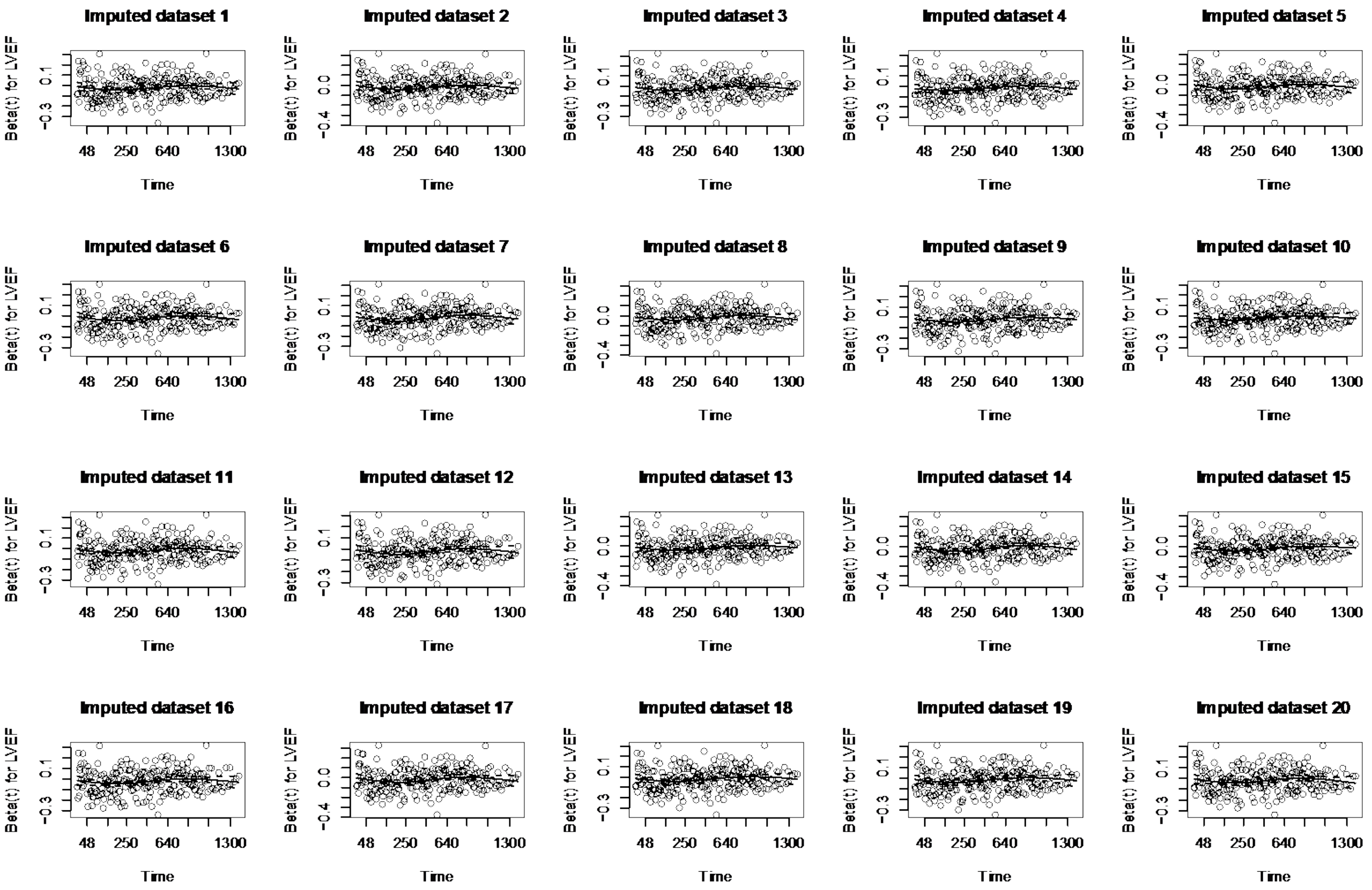

Supplemental Table 14. Model discrimination for the 'original' parsimonious model and for an alternative model using penalised smoothing splines for continuous variables

	Model	
**Imputed dataset**	**Original**	**pspline**
1	0·819	0·828
2	0·821	0·829
3	0·819	0·828
4	0·822	0·831
5	0·820	0·828
6	0·824	0·833
7	0·821	0·829
8	0·822	0·831
9	0·819	0·828
10	0·822	0·830
11	0·820	0·828
12	0·820	0·829
13	0·820	0·827
14	0·822	0·832
15	0·819	0·827
16	0·819	0·828
17	0·821	0·830
18	0·821	0·829
19	0·824	0·833
20	0·825	0·834
**Median**	0·821	0·829

Abbreviations per previous tables.

Supplemental Table 15. Internal validation ('model performance') results for the parsimonious multivariable model

**Imputed dataset**	**Discrimination**	**Optimism adjusted C-index**	**Slope**
1	0·820	0·808	0·929
2	0·821	0·809	0·929
3	0·819	0·807	0·923
4	0·823	0·811	0·931
5	0·820	0·808	0·928
6	0·824	0·8013	0·927
7	0·821	0·809	0·928
8	0·823	0·811	0·927
9	0·820	0·808	0·929
10	0·823	0·812	0·932
11	0·821	0·808	0·927
12	0·822	0·810	0·928
13	0·820	0·809	0·933
14	0·824	0·812	0·928
15	0·819	0·807	0·933
16	0·819	0·807	0·924
17	0·823	0·810	0·924
18	0·822	0·811	0·930
19	0·825	0·813	0·931
20	0·827	0·815	0·929
**Median**	0·821	0·810	0·928

Supplemental Figure 9. Calibration plot of bootstrap resampling estimates of predicted probability of hospitalisation for heart failure or all-cause mortality at 3-years versus observed probabilities using the flexible hazard regression approach[5,6]

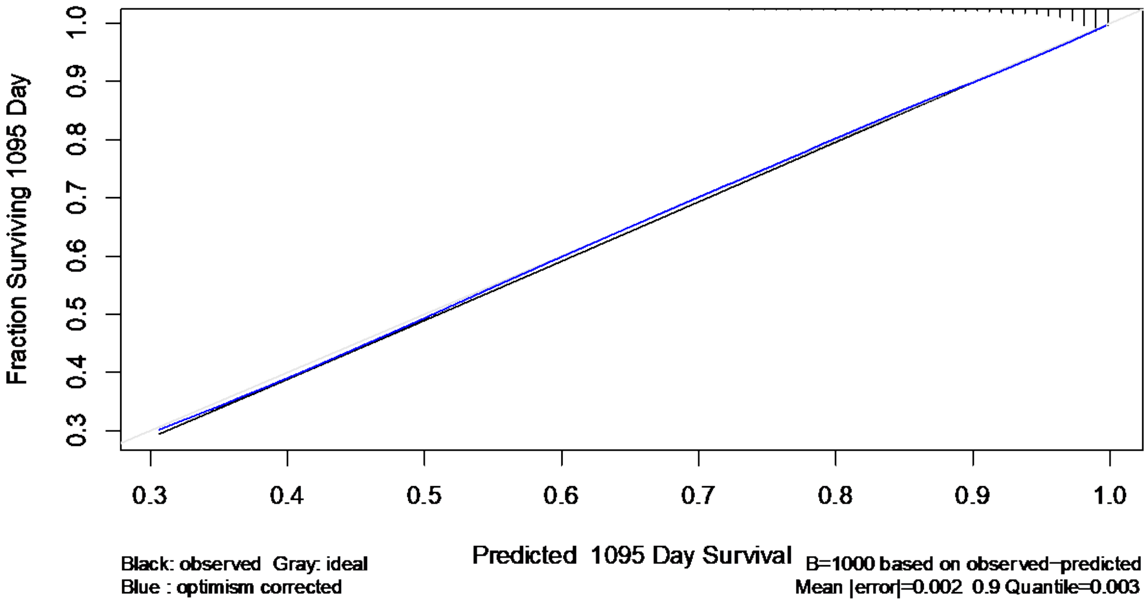

The smooth black line is the apparent calibration, and the blue line is the bootstrap optimism- (overfitting-) corrected calibration curve, both estimated by adaptive linear spline hazard regression. The grey line is the line of identity and represents perfect calibration. Mean |error| is equivalent to the ICI and 0.9 quantile is equivalent to E_{90}.[7] A rug plot of the distribution of predicted outcome probabilities sits on the top axis of the plot. Survival is survival free of hospitalisation for heart failure or all-cause mortality.

Supplemental Table 16. Optimism-adjusted pooled model coefficients for the externally validated parsimonious multivariable model for time to hospitalisation for heart failure or all-cause mortality

	**Hazard ratio**	**95% confidence interval**	**p value**
Age	1·029	1·017 - 1·041	<0·0001
Diabetes	1·480	1·109 - 1·975	0·0079
COPD	2·124	1·510 – 2·989	<0·0001
Ln (NT-proBNP)	1·255	1·102 - 1·430	0·0008
LVEF	0·974	0·963 - 0·983	<0·0001
Infarct LGE	1·521	1·165 - 1·986	0·0022
ECV	1·076	1·037 - 1·117	0·0001

Abbreviations per previous tables.

Supplemental Table 17. Unadjusted pooled model coefficients for the externally validated parsimonious multivariable model for time to hospitalisation for heart failure or all-cause mortality

	**Hazard Ratio**	**95% confidence interval**	**Wald χ^2**	**p value**
Age	1·031	1·018 - 1·043	23·696	<0·0001
Diabetes	1·516	1·116 - 2·058	7·188	0·0079
COPD	2·224	1·548 - 3·194	18·928	<0·0001
Ln (NT-proBNP)	1·273	1·108 - 1·462	11·977	0·0008
LVEF	0·972	0·961 - 0·983	24·642	<0·0001
Infarct LGE	1·560	1·175 - 2·071	9·587	0·0022
ECV	1·081	1·040 - 1·124	15·482	0·0001

Abbreviations per previous tables.

Supplemental Table 18. Univariable and model Schöenfeld test results for the externally validated parsimonious model in 20 imputed datasets, pooled with the D_2 method for χ^2 values

	Imputed dataset																				
	**1**	**2**	**3**	**4**	**5**	**6**	**7**	**8**	**9**	**10**	**11**	**12**	**13**	**14**	**15**	**16**	**17**	**18**	**19**	**20**	**Pooled**
Age	0·60	0·61	0·60	0·59	0·61	0·59	0·58	0·60	0·58	0·56	0·63	0·60	0·57	0·59	0·58	0·58	0·58	0·55	0·55	0·61	0·59
Diabetes	0·52	0·56	0·49	0·49	0·55	0·53	0·54	0·49	0·50	0·49	0·49	0·50	0·50	0·55	0·51	0·51	0·55	0·52	0·53	0·53	0·52
COPD	0·48	0·52	0·49	0·52	0·48	0·52	0·47	0·48	0·49	0·51	0·49	0·51	0·52	0·52	0·52	0·52	0·49	0·52	0·52	0·51	0·50
Ln (NT-proBNP)	0·084	0·017	0·055	0·12	0·032	0·016	0·28	0·048	0·077	0·068	0·009	0·065	0·070	0·11	0·083	0·083	0·022	0·084	0·049	0·0087	0·066
LVEF	0·0053	0·0053	0·0045	0·0061	0·0057	0·0048	0·0047	0·0055	0·0056	0·0074	0·0048	0·0057	0·0048	0·0052	0·0060	0·0060	0·0048	0·0071	0·0050	0·0052	0·0054
Infarct LGE	0·17	0·19	0·17	0·19	0·18	0·17	0·18	0·19	0·20	0·20	0·18	0·18	0·20	0·18	0·18	0·18	0·18	0·19	0·19	0·17	0·18
ECV	0·54	0·11	0·47	0·84	0·39	0·33	0·39	0·28	0·63	0·31	0·48	0·26	0·99	0·73	0·76	0·76	0·54	0·79	0·80	0·14	0·53
**Multivariable model**	0·066	0·10	0·14	0·16	0·15	0·09	0·18	0·16	0·17	0·19	0·069	0·18	0·14	0·18	0·16	0·16	0·10	0·17	0·12	0·077	0·10

Abbreviations per previous tables.

Supplemental Figure 10. Scaled Schöenfeld residual plots for LV ejection fraction against time in the 20 imputed datasets for the externally validated model

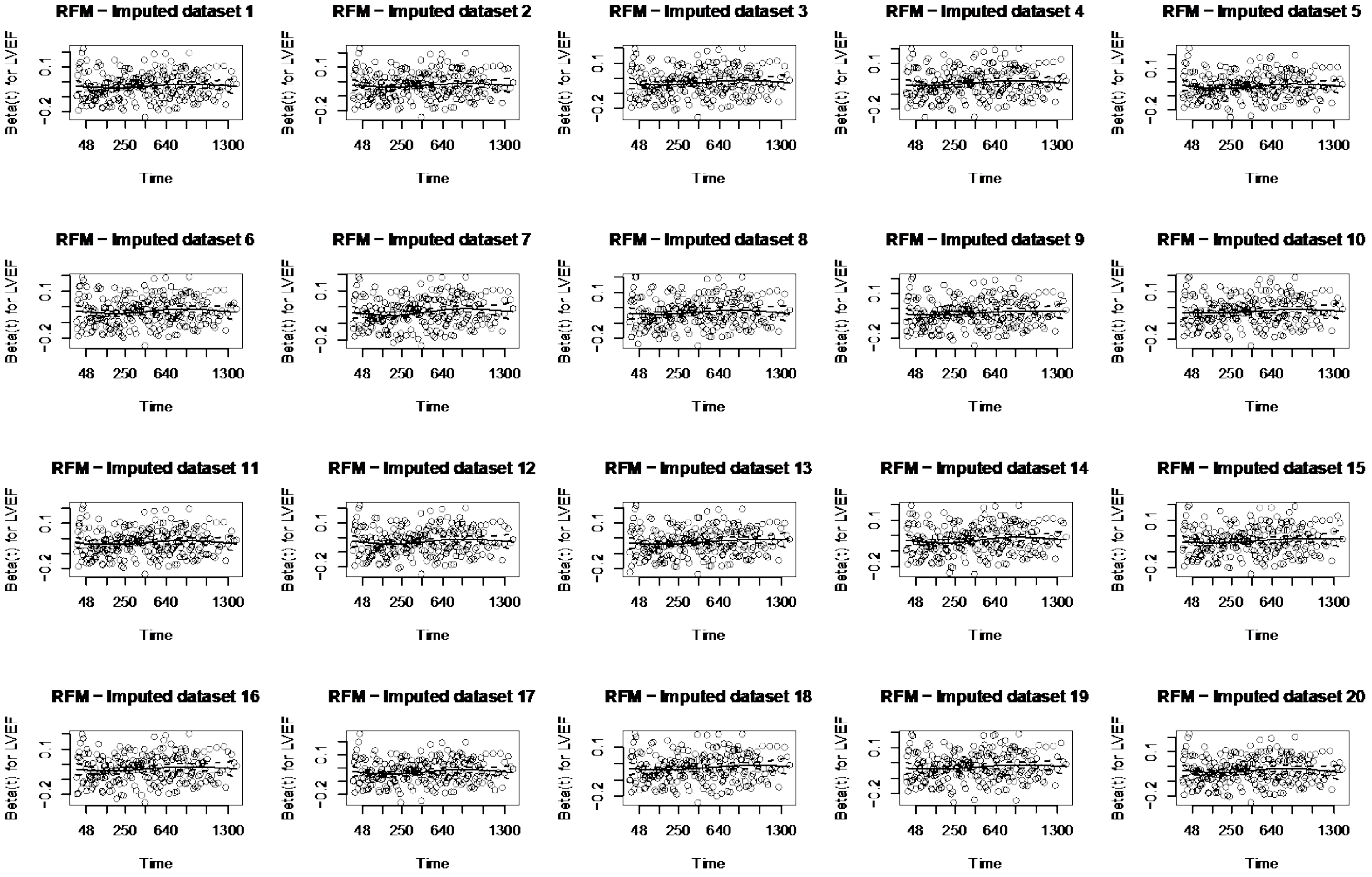

Supplemental Table 19. Model discrimination for the ‘original’ externally validated parsimonious model and an alternative model using penalised smoothing splines for continuous covariables

	**Model**	
**Imputed dataset**	**Original**	**pspline**
1	0·816	0·822
2	0·818	0·824
3	0·817	0·823
4	0·819	0·825
5	0·819	0·824
6	0·822	0·827
7	0·819	0·825
8	0·819	0·825
9	0·817	0·823
10	0·820	0·825
11	0·817	0·823
12	0·818	0·823
13	0·818	0·824
14	0·820	0·827
15	0·816	0·822
16	0·819	0·823
17	0·819	0·825
18	0·819	0·824
19	0·822	0·827
20	0·822	0·828
Median	0·819	0·824

Abbreviations per previous tables.

Supplemental Table 20. Internal validation ('model performance') results for the final, externally validated parsimonious multivariable model

Imputed dataset	Discrimination	Optimism adjusted C-index	Slope
1	0·816	0·806	0·943
2	0·818	0·809	0·943
3	0·817	0·807	0·938
4	0·819	0·810	0·944
5	0·819	0·809	0·941
6	0·822	0·813	0·943
7	0·819	0·809	0·941
8	0·819	0·809	0·942
9	0·817	0·807	0·941
10	0·820	0·811	0·944
11	0·817	0·806	0·941
12	0·818	0·808	0·944
13	0·818	0·809	0·945
14	0·820	0·810	0·941
15	0·816	0·807	0·948
16	0·816	0·806	0·939
17	0·819	0·809	0·939
18	0·819	0·810	0·945
19	0·822	0·812	0·943
20	0·822	0·813	0·943
**Median**	0·819	0·809	0·943

Supplemental Figure 11. Re-calibration plots for the optimism-adjusted multivariable model in the external validation cohort at 1- and 3- years.

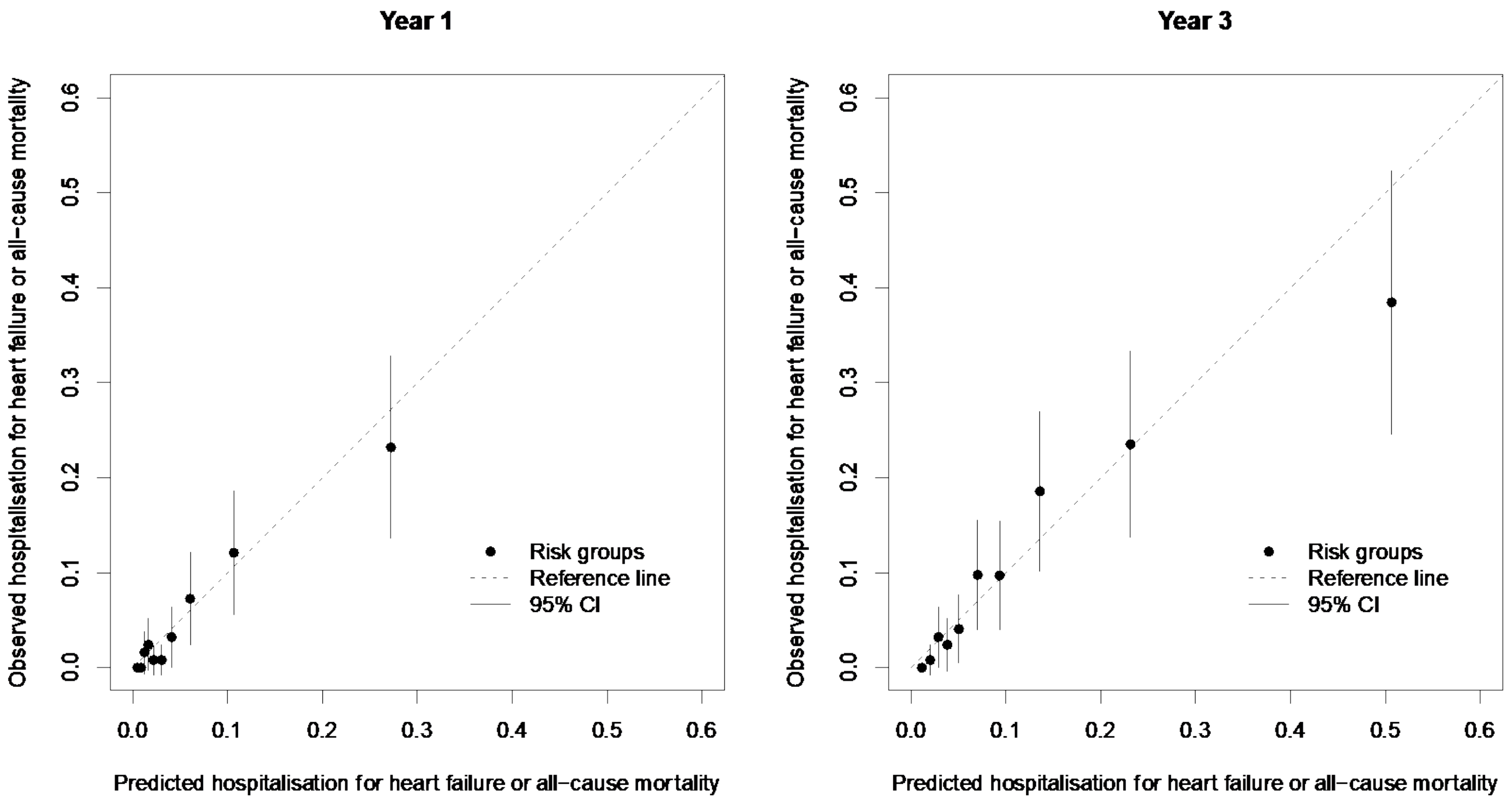

Supplemental Figure 12. Calibration plots prior to baseline hazard updating for the optimism-adjusted multivariable model in the external validation cohort at 1- and 3-years.

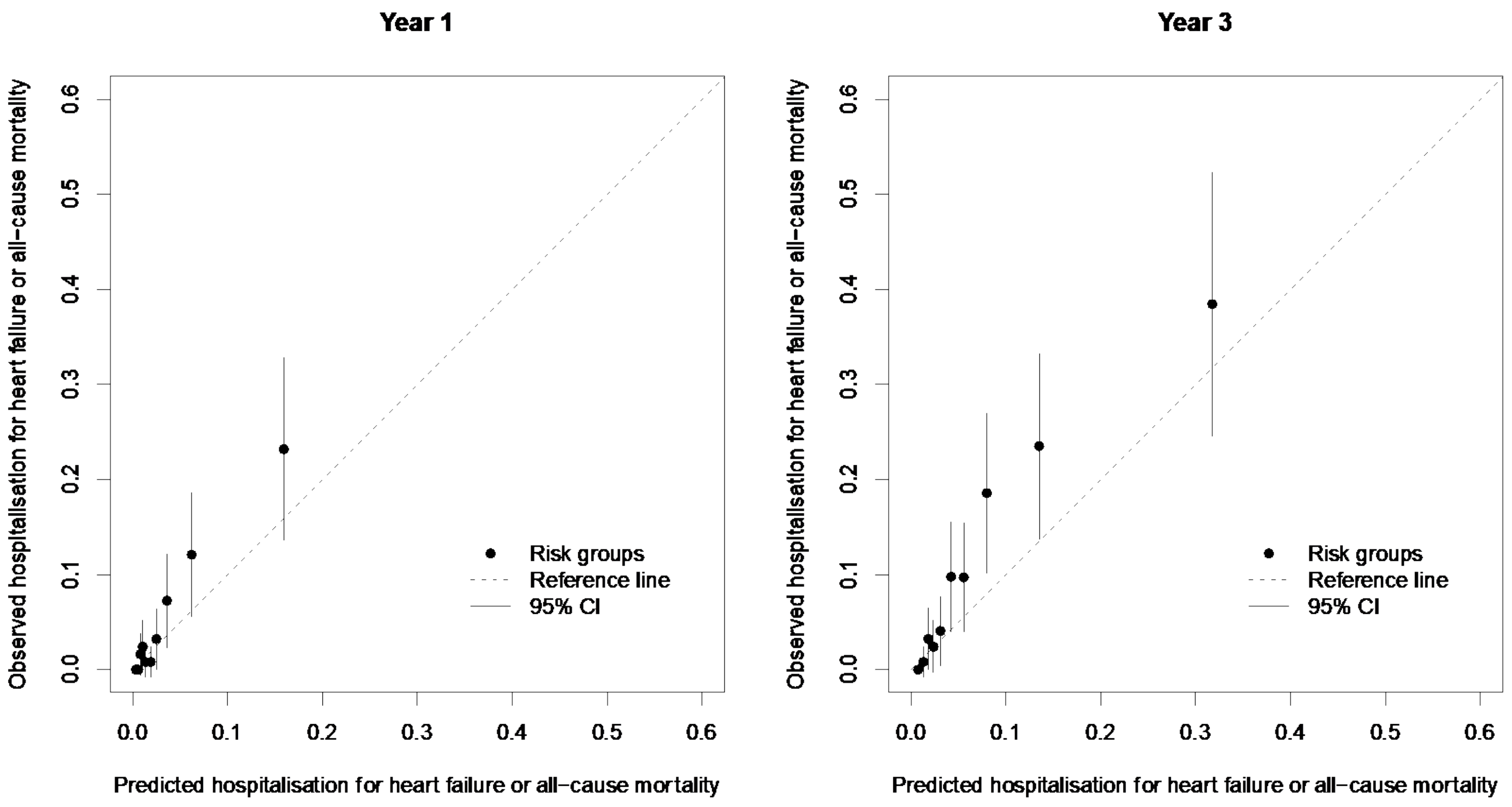

Supplemental Figure 13. Calibration plots prior to baseline hazard updating for the unadjusted multivariable model in the external validation cohort at 1- and 3-years.

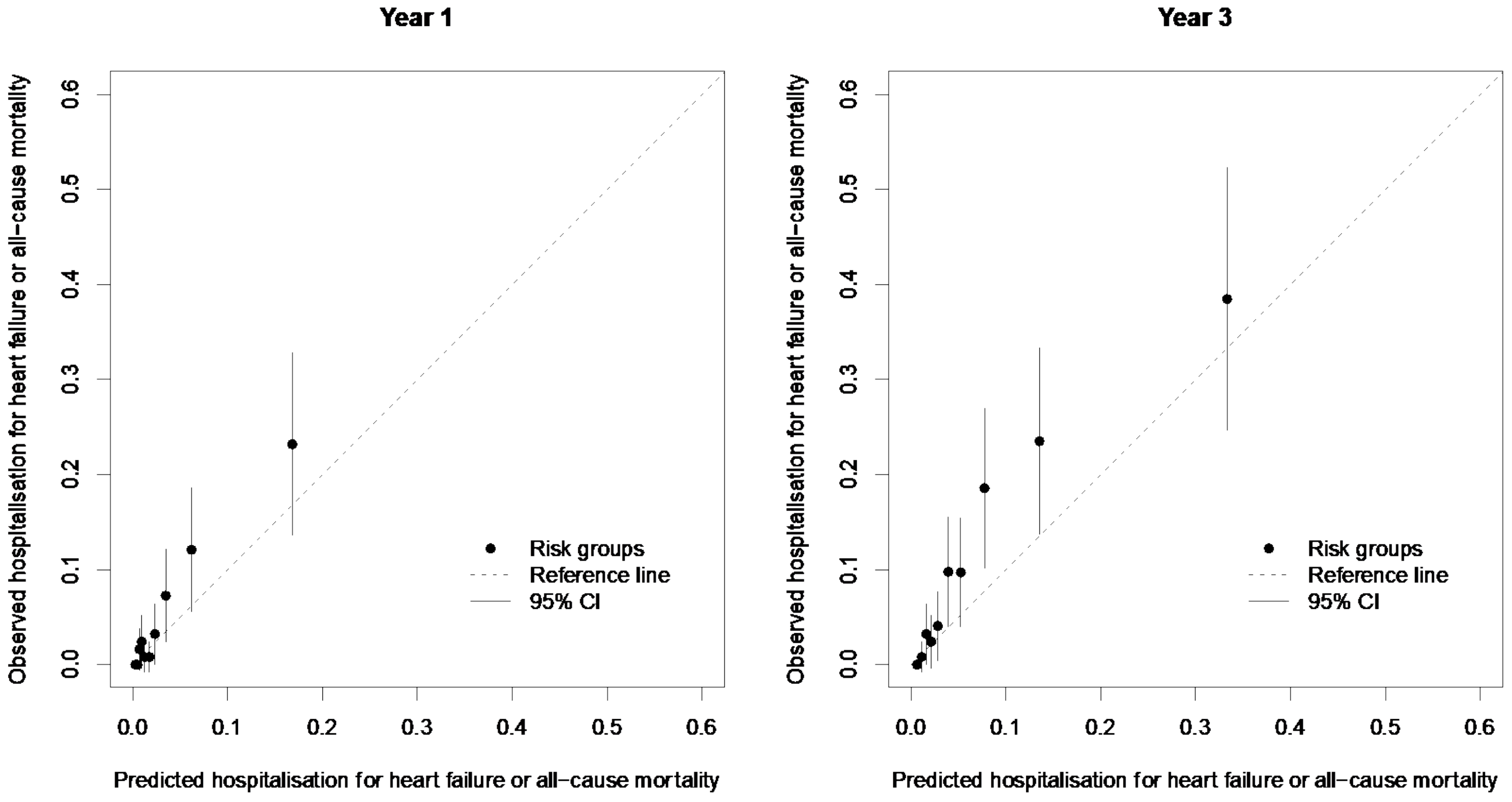

Supplemental Figure 14. Survival free of hospitalisation for heart failure and all-cause mortality in the model development (A) and external validation (B) cohorts, according to predicted probability.

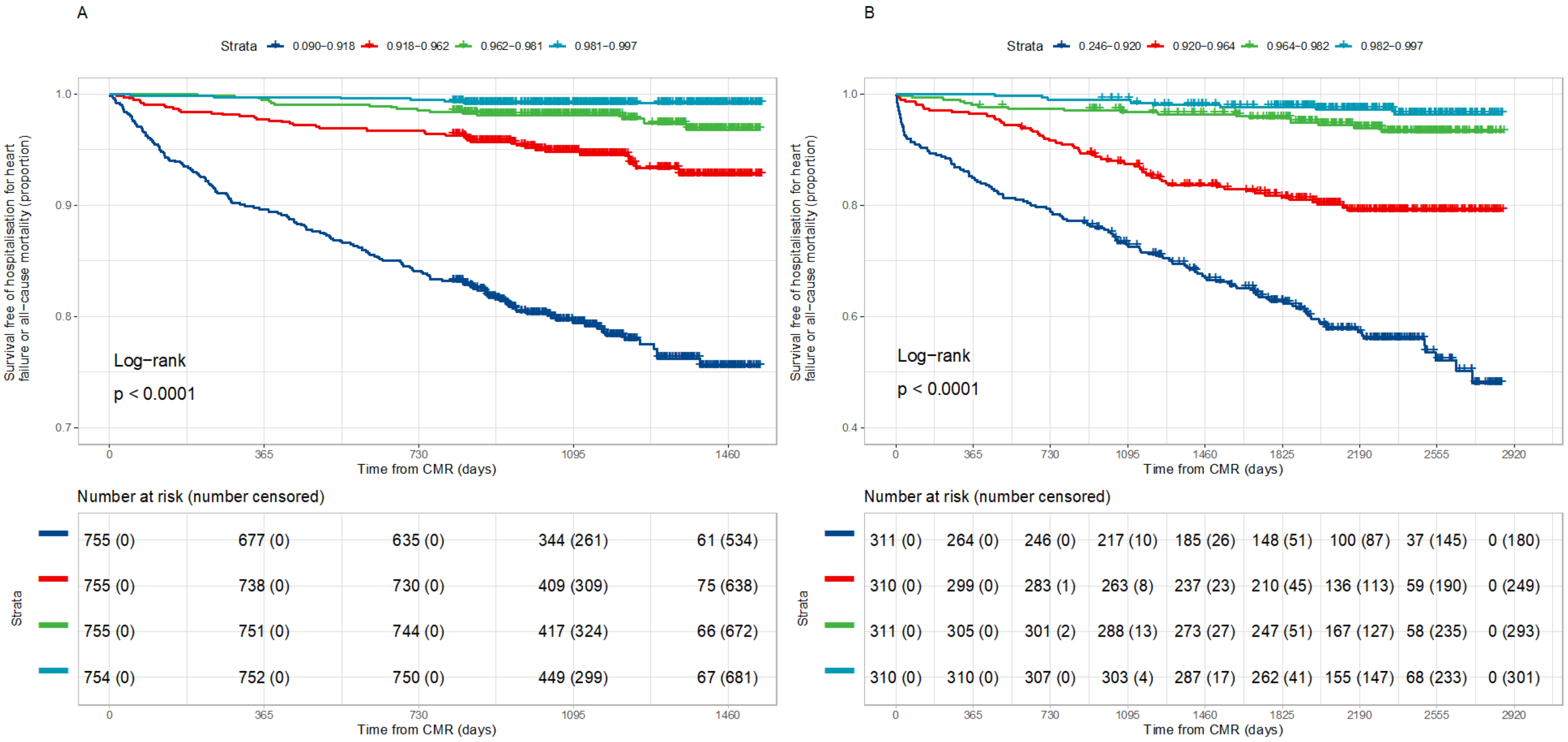

Strata = cohorts were divided into quartiles according to predicted probability.

Supplemental Table 21. Preliminary analysis comparing model discrimination when heart failure was the primary diagnosis versus when heart failure was the primary or secondary diagnosis

	**Outcome (hospitalisation for heart failure or all-cause mortality)**	
	**Using heart failure as the primary diagnosis**	**Using heart failure as the primary or secondary diagnosis**
**C-index (95% CI)**	0·806 (0·789 – 0·828)	0·799 (0·777 – 0·821)
**Events**	225	351

Supplemental Table 22. Comparison of imputed variable for imputation models including the candidate predictors, outcome status variable and time-to-outcome or the Nelson-Aalen estimate

		**Imputation model**				
		**Time-to-outcome**			**Nelson-Aalen**	
**Incomplete Variable**	**% missing**	**Mean**	**standard deviation**		**Mean**	**standard deviation**
Multiple Deprivation Index	1·2	16247·14	10152·46		16257·42	10154·16
Body Mass Index	1·6	28·606588	6·061639		28·60676	6·06943
QRS duration	14·0	103·63331	23·26444		103·69702	23·37149
Estimated glomerular filtration rate	1·1	78·54664	13·03623		78·54435	13·02982
N-terminal pro-B-type natriuretic peptide	22·4	508·2462	1306·1870		507·4117	1315·7745
High sensitivity cardiac troponin T	22·6	21·36277	158·76403		21·68507	158·35024
Left ventricle ejection fraction	0·1	56·21179	12·18117		56·20999	12·18112
Indexed myocardial mass	0·4	58·34136	17·67830		58·34711	17·68250
Global longitudinal strain	3·3	-17·693013	4·465356		-17·694651	4·464198
Right ventricle ejection fraction	0·2	56·505532	9·366426		56·503279	9·365461
Indexed left atrial area	1·0	14·143806	3·516133		14·142751	3·516653
Myocardial extracellular volume	12·6	26·065318	3·327803		26·068372	3·332822

Variables where no imputation was required are not presented.

Supplemental Figure 15. Correlations between candidate predictors

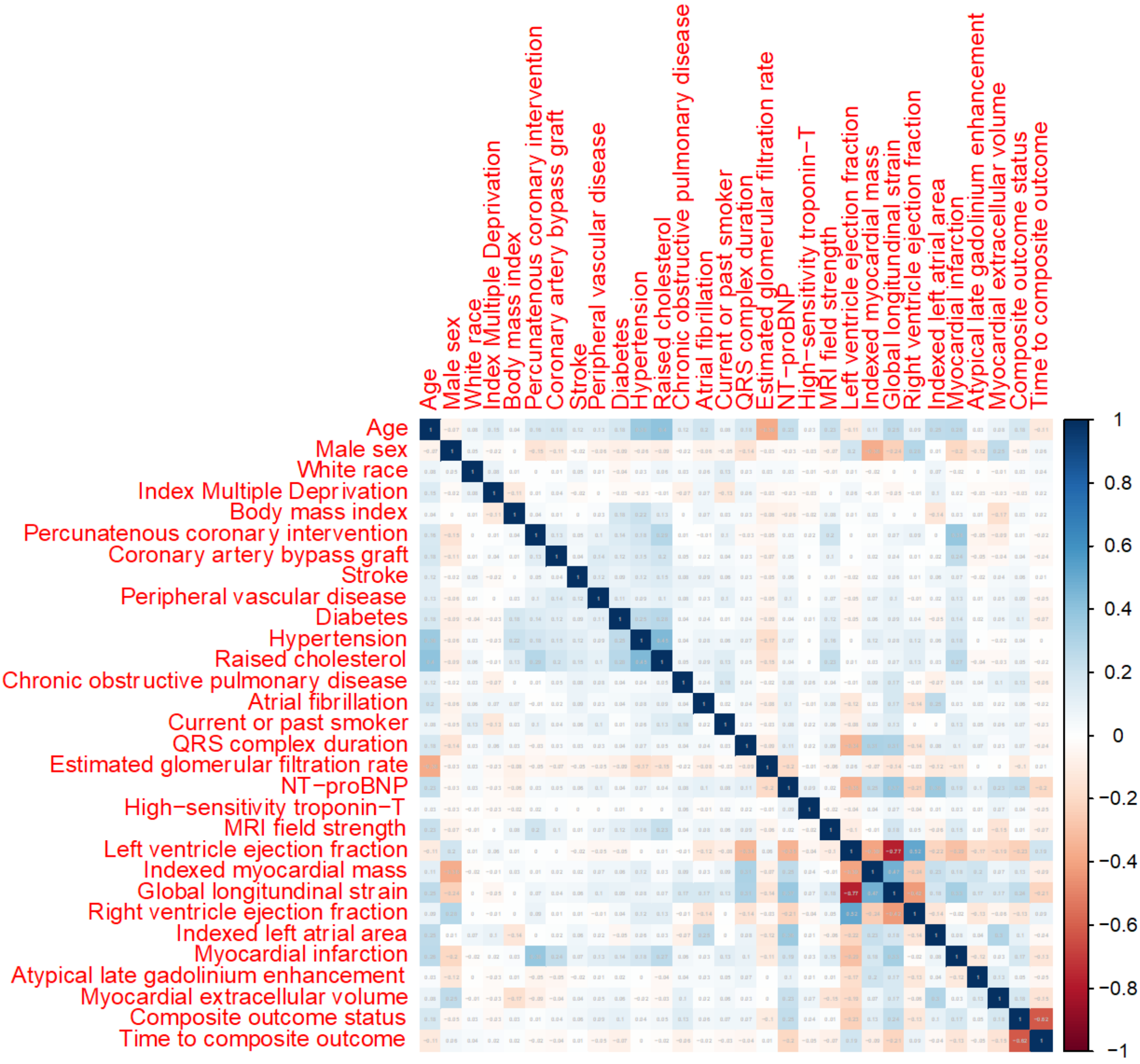

Further analyses

The following results are not described in the main manuscript but are included for additional information.

Model performance by heart failure stage

A preliminary evaluation was conducted to compare model discrimination in subgroups of patients with stage A and B vs C and D heart failure in the derivation cohort, using a complete case analysis (Supplemental Table 23). Model discrimination appears high in both groups, albeit marginally higher in stage A and B.

Supplemental Table 23. Preliminary comparison of model discrimination for patients with Stage A/B heart failure versus Stage C/D

	**Heart failure stage**	
	**A/B**	**C/D**
**n**	1059	947
**C-index (95% CI)**	0·814 (0·747 – 0·881)	0·775 (0·732 – 0·818)
**Events**	40	105

Complete cases only

References

1. Klersy C, d'Eril GVM, Barassi A, et al. Advantages of the lognormal approach to determining reference change values for N-terminal propeptide B-type natriuretic peptide. *Clinica Chimica Acta* 2012; **413**(5-6): 544-7.
2. Marshall A, Altman DG, Holder RL, Royston P. Combining estimates of interest in prognostic modelling studies after multiple imputation: current practice and guidelines. *BMC Medical Research Methodology* 2009; **9**(1): 57.
3. Enders CK. Applied missing data analysis. New York: Guilford Press; 2010.
4. Eilers PHC, Marx BD. Flexible smoothing with B-splines and penalties. *Statistical Science* 1996; **11**(2): 89-121.
5. Kooperberg C, Stone CJ, Truong YK. Hazard Regression. *Journal of the American Statistical Association* 1995; **90**(429): 78-94.
6. Harrell , Frank E. Regression Modeling Strategies: With Applications to Linear Models, Logistic and Ordinal Regression, and Survival Analysis. Cham: Springer International Publishing; 2015.
7. Austin PC, Harrell FE, Klaveren D. Graphical calibration curves and the integrated calibration index (ICI) for survival models. *Statistics in Medicine* 2020; **39**(21): 2714-42.
8. White IR, Royston P. Imputing missing covariate values for the Cox model. *Statistics in Medicine* 2009; **28**(15): 1982-98.
